# Supplementary material for: Machine learning links T cell function and spatial localization to neoadjuvant immunotherapy and clinical outcome in pancreatic cancer
Source: bioRxiv. 2023 Oct 23:2023.10.20.563335. Preprint. [Version 1] doi: 10.1101/2023.10.20.563335 (PMC10634700; doi:10.1101/2023.10.20.563335)
Supplement: 1 [file NIHPP2023.10.20.563335V1-supplement-1.pdf]

bioRxiv preprint doi: https://doi.org/10.1101/2023.10.20.563335; this version posted October 23, 2023. The copyright holder for this preprint (which was not certified by peer review) is the author/funder, who has granted bioRxiv a license to display the preprint in perpetuity. It is made available under aCC-BY 4.0 International license.

SUPPLEMENTARY FIGURE LEGENDS

Supplementary Figure S1.

A. Representative IHC staining of each antibody used in sequence in the panel. Scale bar = 50 μm.

B. Two representative regions stained with CD3, CD8, and CD4 antibodies. For each region, top images show gates for CD8 on $CD3^+$ population (left) and CD4 on $CD3^+$ $CD8^-$ population (right), and bottom row shows pseudo-colored mIHC images.

C. Hierarchical gating template used to phenotype cells using image gating cytometry in FCS Image Cytometry RUO.

Supplementary Figure S2.

A. SHAP plots showing the top 30 features driving each histopathologic model. Features are ordered on the y-axis such that those with a larger impact on model's predictions appear at the top of the SHAP plots. SHAP values are shown on the x-axis, with a value of zero (center) indicating no impact on the model, and negative or positive SHAP values predicting treatment-naive or αCD40-treated tissues, respectively. Red or blue dots indicate presence or absence, respectively, of the corresponding feature in the tissue.

B-E. Box plots showing feature values for each of the top 15 features for models derived from T, IA, TAS, or NAP sites, respectively, split by treatment cohort. Each dot represents the log10+1 normalized feature value for one tissue region, inputted into the classifier model. Boxes = quartile 1 (Q1) to quartile 3 (Q3); whiskers = smallest and largest datapoints within 1.5*interquartile range (IQR) +/- Q3/Q1;

bioRxiv preprint doi: https://doi.org/10.1101/2023.10.20.563335; this version posted October 23, 2023. The copyright holder for this preprint (which was not certified by peer review) is the author/funder, who has granted bioRxiv a license to display the preprint in perpetuity. It is made available under aCC-BY 4.0 International license.

solid line = median. Mann–Whitney *U*-test used to determine statistical significance. *P*-values corrected using the Benjamini–Hochberg procedure. *, $P \leq 0.05$; **, $P \leq 0.01$; ***, $P \leq 0.001$.

B. T site, n= 55 treatment-naive and n = 48 αCD40-treated regions per feature.

C. IA site, n= 89 treatment-naive and n = 43 αCD40-treated regions per feature.

D. TAS site, n = 25 treatment-naive and n = 27 αCD40-treated regions per feature.

E. NAP site, n = 6 treatment-naive and n = 13 αCD40-treated regions per feature.

Supplementary Figure S3.

A. SHAP plot showing the top 30 features driving the IA model. Features are ordered on the y-axis such that those with a larger impact on the model's predictions appear at the top of the SHAP plot. SHAP values are shown on the x-axis, with a value of zero (center) indicating no impact on the model, and negative or positive SHAP values predicting long DFS or short DFS, respectively. Red or blue dots indicate presence or absence, respectively, of the corresponding feature in tissues.

B. Box plot showing feature values for each of the top 15 features for the model derived from IA regions of the αCD40 cohort split by DFS group (n = 30 regions from short DFS patients per feature; n = 13 regions from long DFS patients per feature). Each dot represents the log10+1 normalized feature value for one tissue region, which was inputted into the classifier model. Boxes = Q1 to Q3; whiskers = smallest and largest datapoints within 1.5*IQR +/- Q3/Q1; solid line = median. Mann–Whitney *U*-test used to determine statistical significance. *P*-values

bioRxiv preprint doi: https://doi.org/10.1101/2023.10.20.563335; this version posted October 23, 2023. The copyright holder for this preprint (which was not certified by peer review) is the author/funder, who has granted bioRxiv a license to display the preprint in perpetuity. It is made available under aCC-BY 4.0 International license.

corrected using the Benjamini–Hochberg procedure. *, $P \leq 0.05$; **, $P \leq 0.01$; ***, $P \leq 0.001$.

Supplementary Figure S4.

A. Elbow plot showing optimal number of RCNs (k=7) for grouping cellular neighborhoods.

B. Bar chart showing the number of cells assigned to each of the seven RCNs across all αCD40 IA regions.

C. Bar chart showing the percentage (out of 100) of cells assigned to each of the seven RCNs across all αCD40 IA regions.

D. Stacked bar chart showing fraction (out of 1.0) of RCNs present per αCD40 IA region.

E. Stacked bar chart showing average proportion (out of 1.0) of RCNs present in IA regions for each αCD40-treated patient.

F. Scatterplot reconstructions for each αCD40 IA region. Each dot represents a cell present in the IA, and each cell is colored by its original cell state phenotype (top scatterplot) or RCN assignment (bottom scatterplot).

SUPPLEMENTARY TABLE LEGENDS

Supplementary Table S1.

Statistical comparison between the Liudahl et al. original PDAC cohort(35) and the selected subset used as Cohort 1 in this study. Mean value and standard error of the

bioRxiv preprint doi: https://doi.org/10.1101/2023.10.20.563335; this version posted October 23, 2023. The copyright holder for this preprint (which was not certified by peer review) is the author/funder, who has granted bioRxiv a license to display the preprint in perpetuity. It is made available under aCC-BY 4.0 International license.

mean (SEM) shown for each variable. *P*-values computed using Fisher's exact test for categorical variables, Wilcoxon rank-sum test for continuous variables, and log rank test for overall survival.

Supplementary Table S2.

Table of antibodies used in mIHC panel.

Supplementary Table S3.

Raw counts of cell states defined by mIHC gating strategy present in the dataset.

Supplementary Table S4.

Raw counts of T cells expressing each functionality barcode present in the dataset.

Supplementary Table S5.

Raw counts of cell-cell spatial interactions present in the dataset.

bioRxiv preprint doi: https://doi.org/10.1101/2023.10.20.563335; this version posted October 23, 2023. The copyright holder for this preprint (which was not certified by peer review) is the author/funder, who has granted bioRxiv a license to display the preprint in perpetuity. It is made available under aCC-BY 4.0 International license.

Blise et al. Figure S1

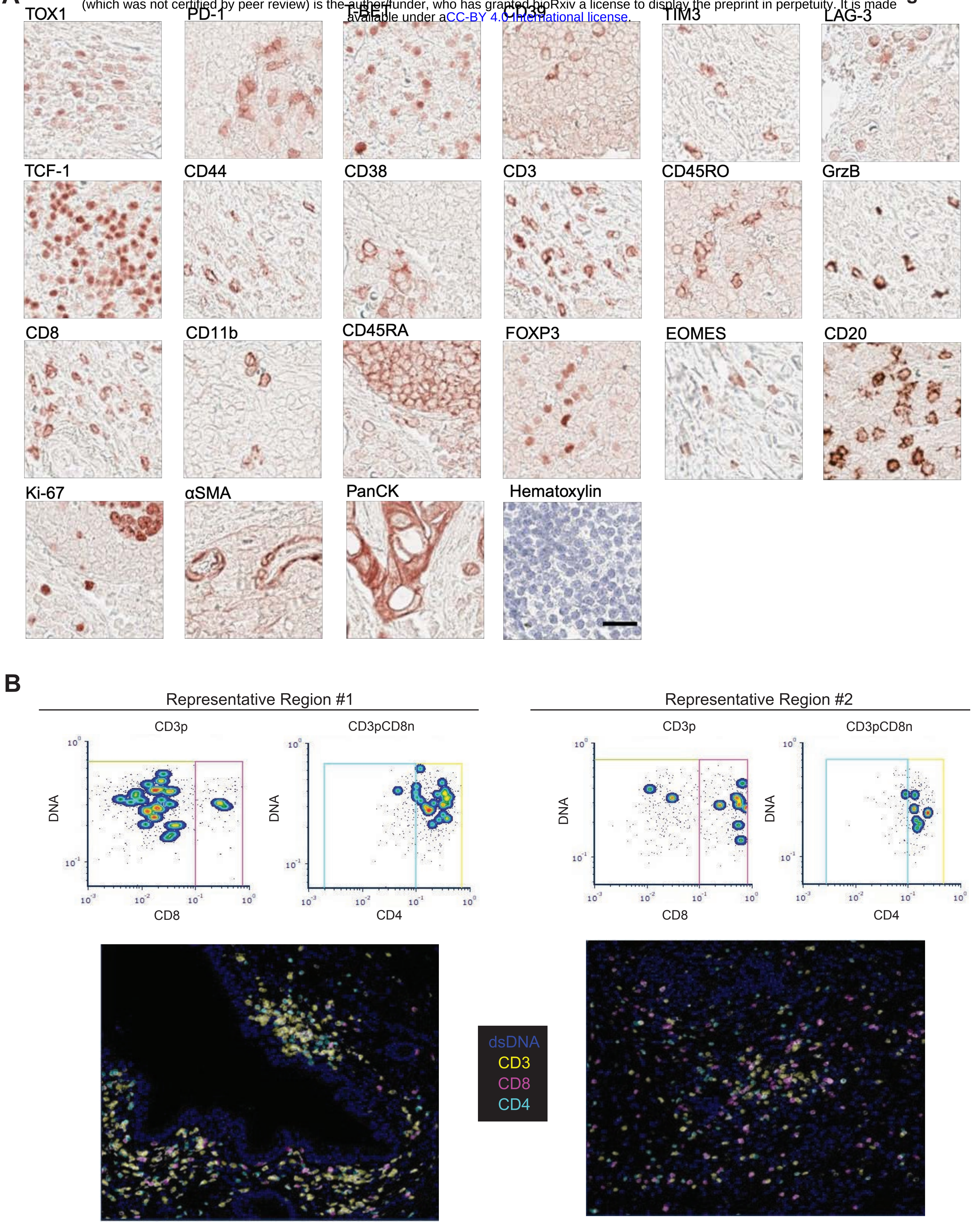

bioRxiv preprint doi: https://doi.org/10.1101/2023.10.20.563335; this version posted October 23, 2023. The copyright holder for this preprint (which was not certified by peer review) is the author/funder, who has granted bioRxiv a license to display the preprint in perpetuity. It is made available under aCC-BY 4.0 International license.

Bliss et al. Figure S1

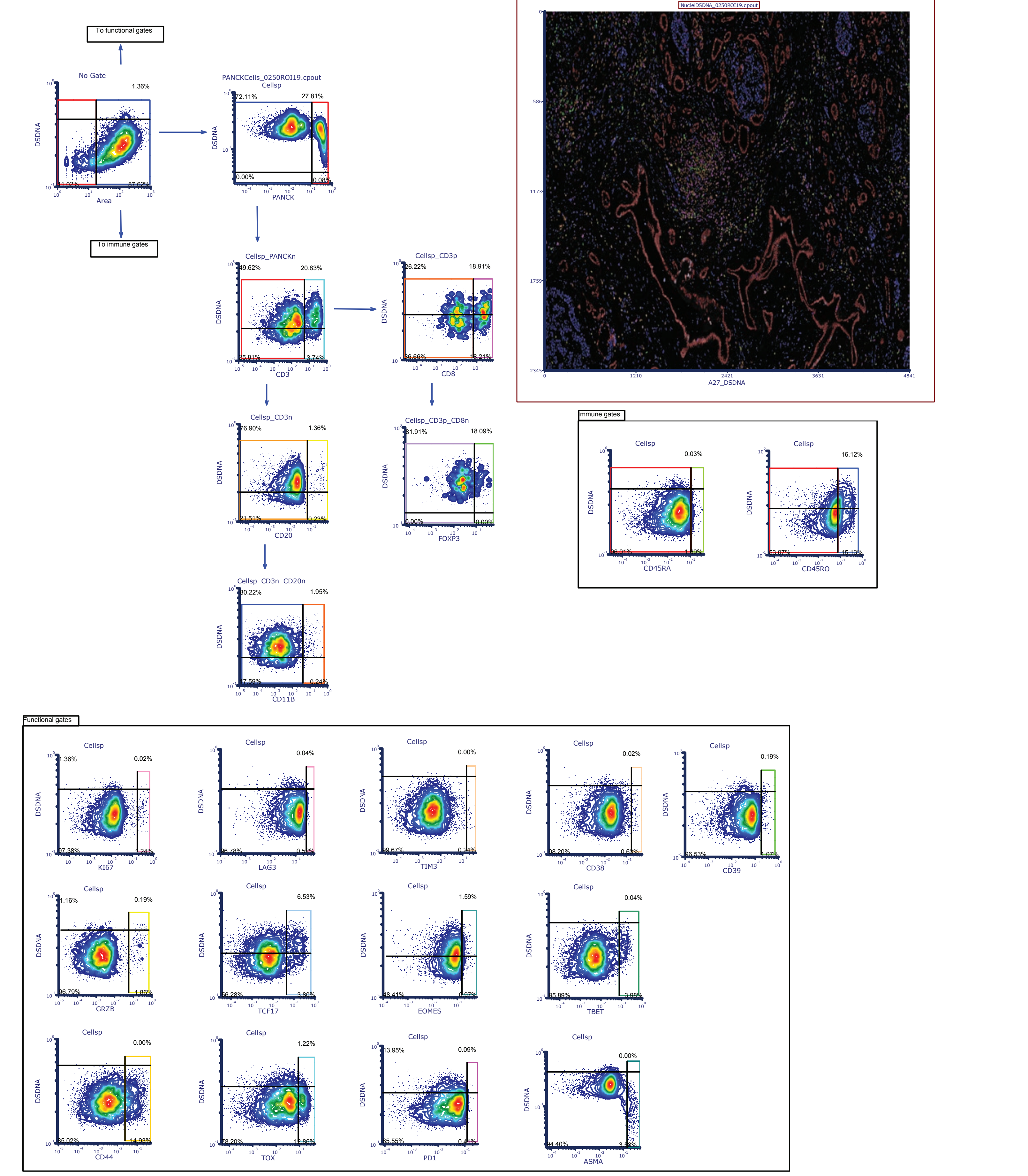

bioRxiv preprint doi: https://doi.org/10.1101/2023.10.20.563335; this version posted October 23, 2023. The copyright holder for this preprint (which was not certified by peer review) is the author/funder, who has granted bioRxiv a license to display the preprint in perpetuity. It is made available under aCC-BY 4.0 International license.

A

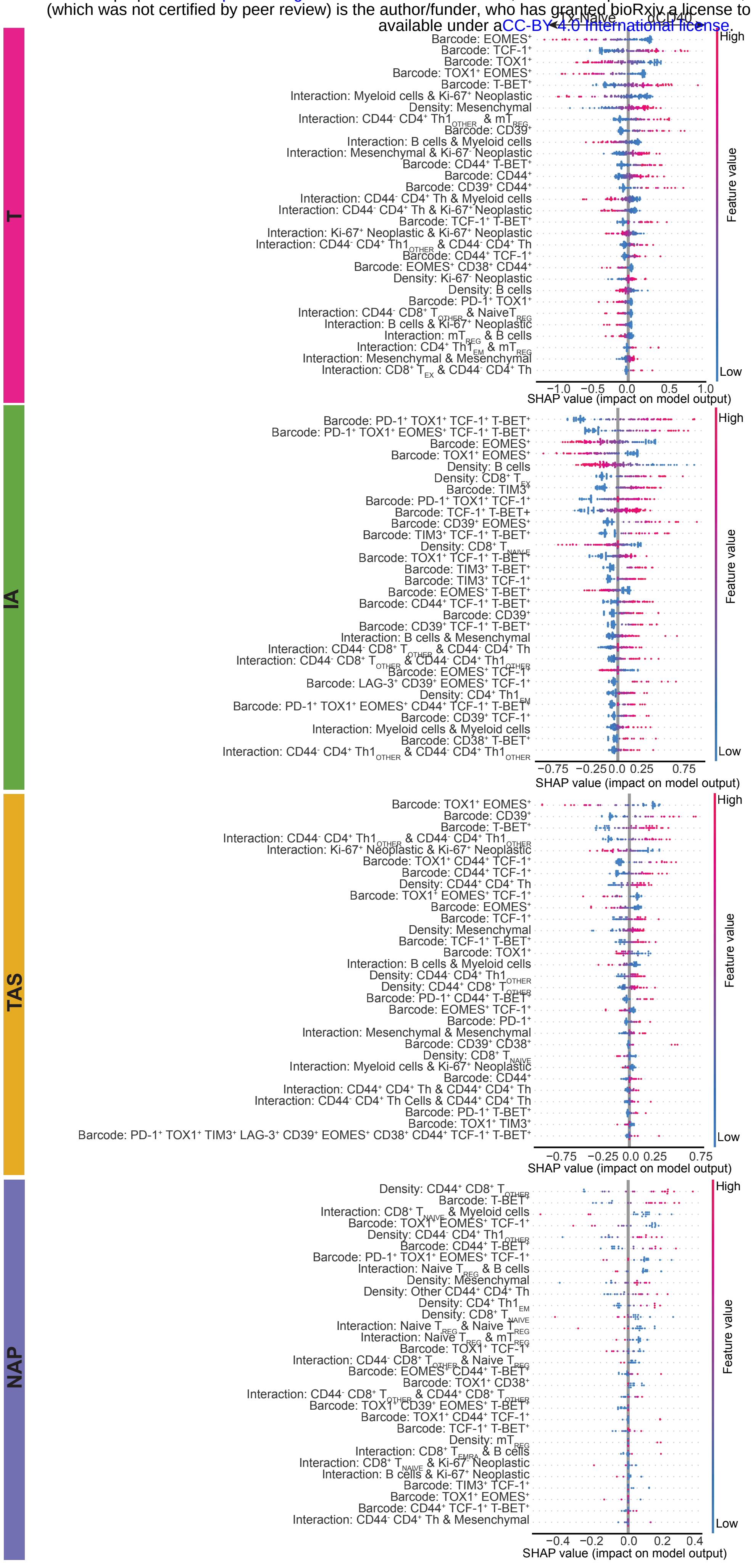

bioRxiv preprint doi: https://doi.org/10.1101/2023.10.20.563335; this version posted October 23, 2023. The copyright holder for this preprint (which was not certified by peer review) is the author/funder, who has granted bioRxiv a license to display the preprint in perpetuity. It is made available under aCC-BY 4.0 International license.

Blise et al. Figure S2

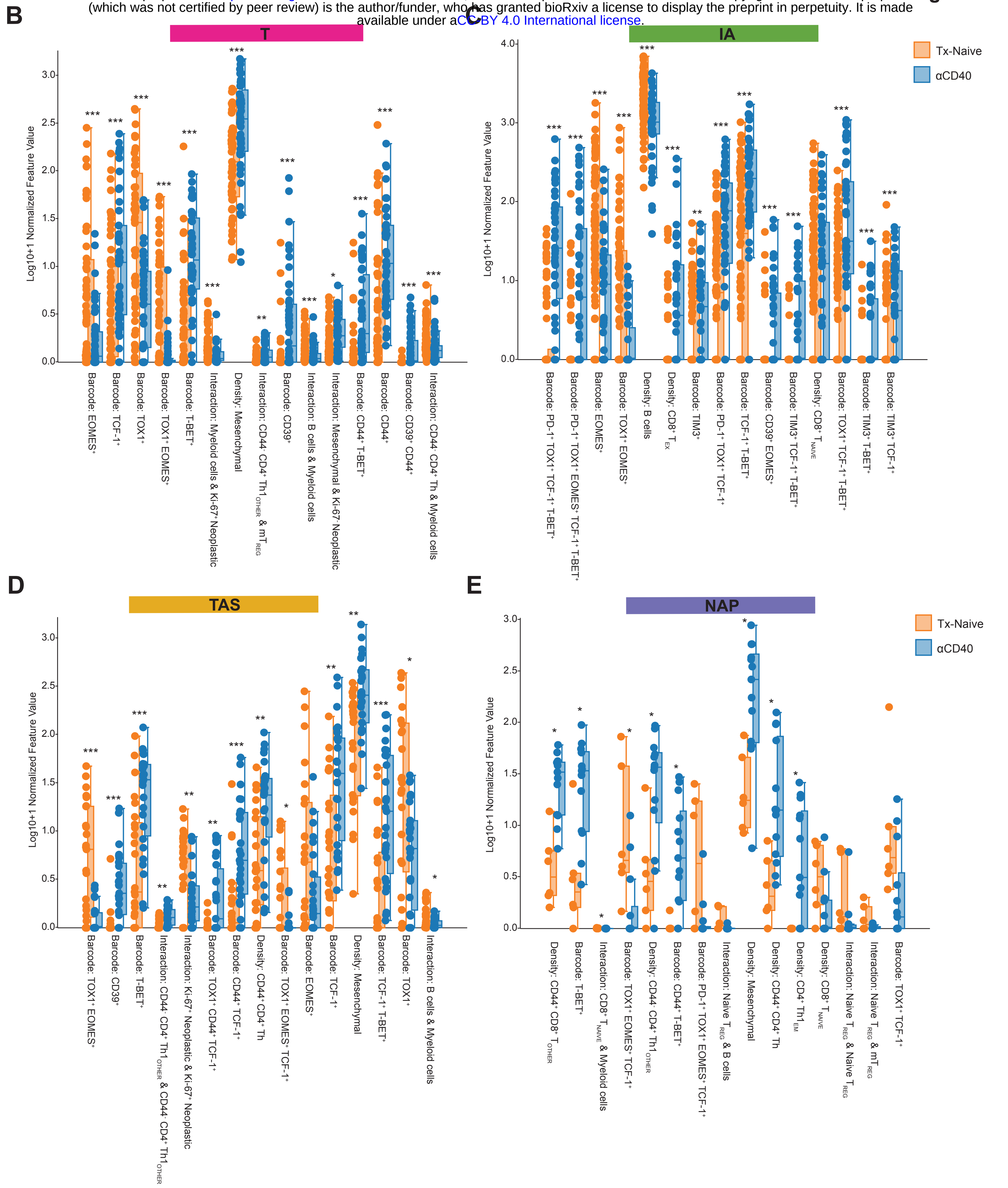

bioRxiv preprint doi: https://doi.org/10.1101/2023.10.20.563335; this version posted October 23, 2023. The copyright holder for this preprint (which was not certified by peer review) is the author/funder, who has granted bioRxiv a license to display the preprint in perpetuity. It is made available under aCC-BY 4.0 International license.

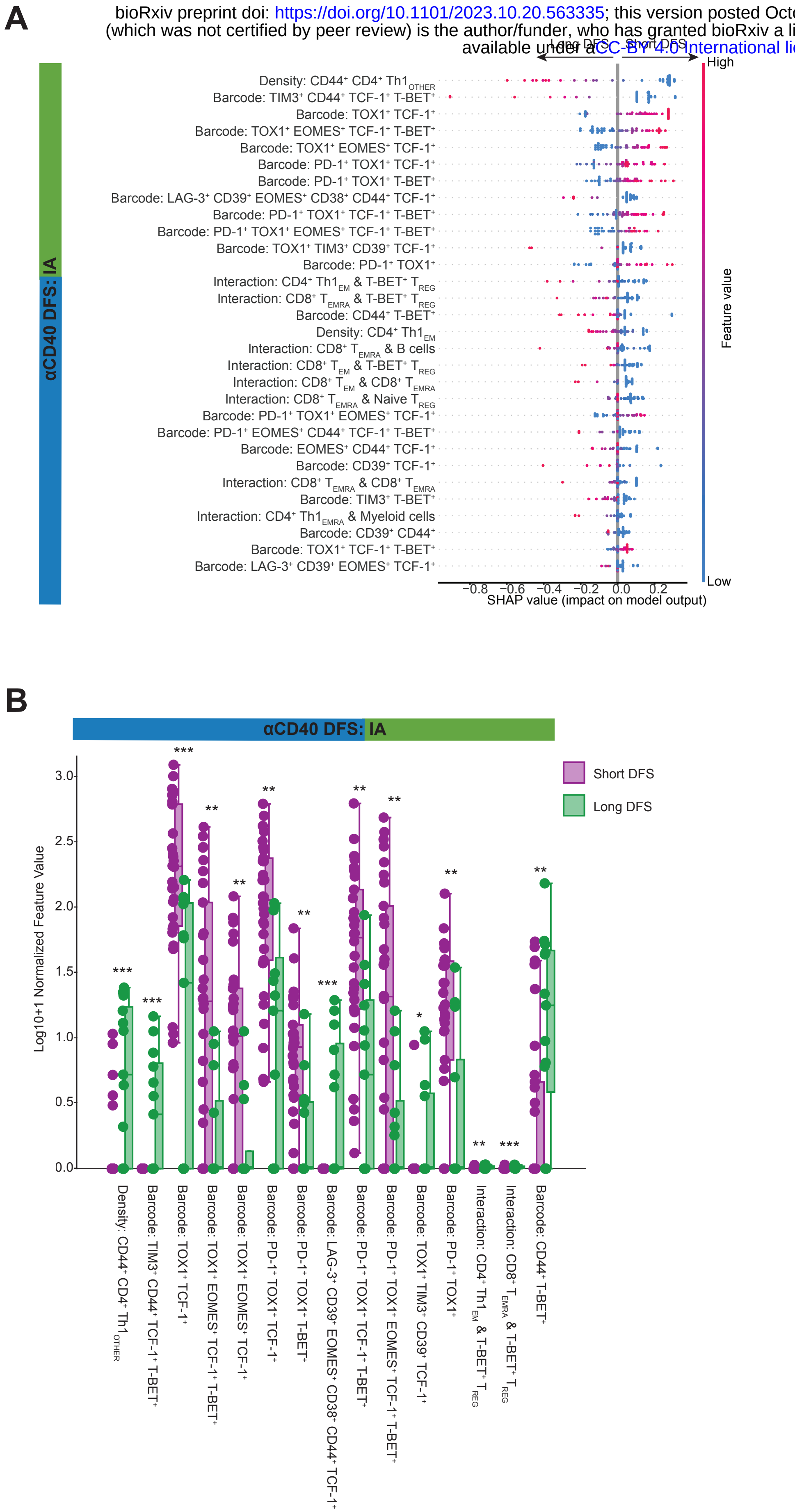

bioRxiv preprint doi: https://doi.org/10.1101/2023.10.20.563335; this version posted October 23, 2023. The copyright holder for this preprint (which was not certified by peer review) is the author/funder, who has granted bioRxiv a license to display the preprint in perpetuity. It is made available under aCC-BY 4.0 International license.

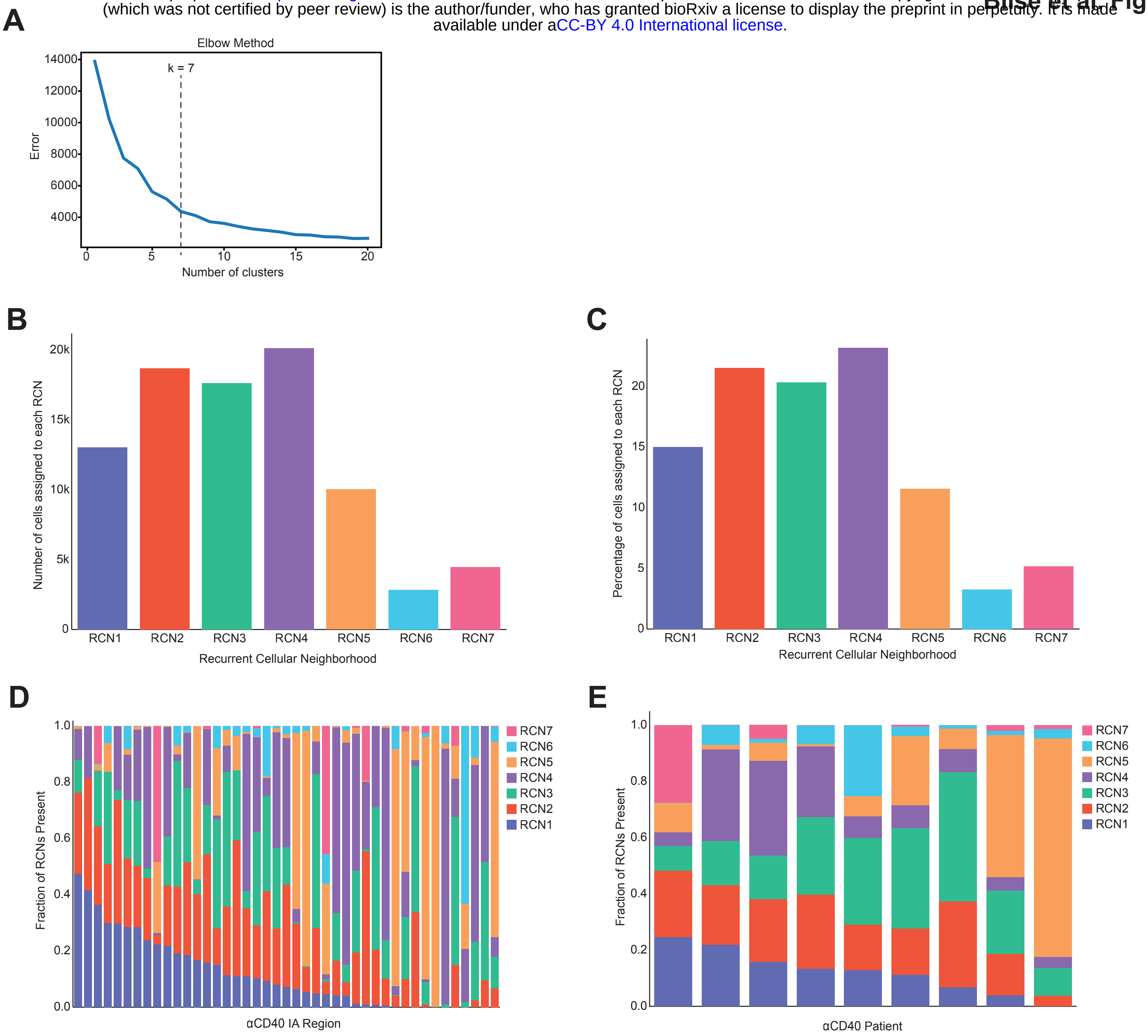

bioRxiv preprint doi: https://doi.org/10.1101/2023.10.20.563335; this version posted October 23, 2023. The copyright holder for this preprint (which was not certified by peer review) is the author/funder, who has granted bioRxiv a license to display the preprint in perpetuity. It is made available under aCC-BY 4.0 International license.

Blise et al. Figure S4

F

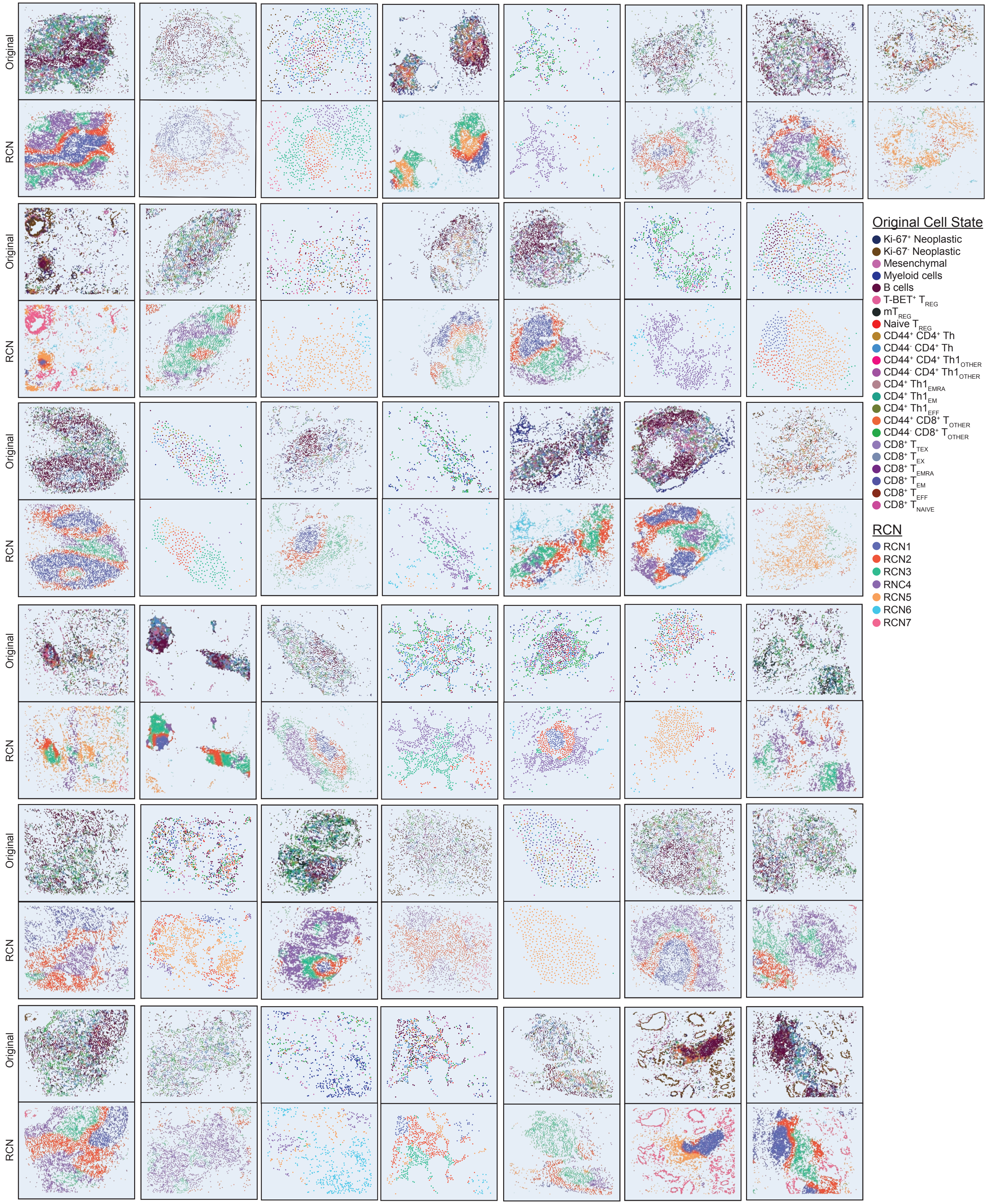

bioRxiv preprint doi: https://doi.org/10.1101/2023.10.20.563335; this version posted October 23, 2023. The copyright holder for this preprint (which was not certified by peer review) is the author/funder, who has granted bioRxiv a license to display the preprint in perpetuity. It is made available under aCC-BY 4.0 International license.

Supplementary Table 1. Statistical comparison between the Liudahl et al. original PDAC cohort and the selected subset used as Cohort 1 in this study. Mean value and standard error of the mean (SEM) shown for each variable. *P*-values computed using Fisher's exact test for categorical variables, Wilcoxon rank-sum test for continuous variables, and log rank test for overall survival.

	**Original Cohort**	**Selected Subset**	***P***
OS (months) (mean ± SEM)	27.34 ± 2.25	27.27 ± 4.75	0.90
# cases OHSU/DFCI	45/59	6/12	0.61
$CD8^+$ T cell density (mean ± SEM)	144.57 ± 11.49	152.51 ± 26.35	0.63
$CD4^+$ T cell density (mean ± SEM)	185.95 ± 12.45	215.15 ± 30.41	0.33
$CD20^+$ B cell density (mean ± SEM)	98.01 ± 10.68	97.73 ± 21.11	0.85
Plasmablast density (mean ± SEM)	12.13 ± 2.13	10.78 ± 2.86	0.89
Plasma cell density (mean ± SEM)	3.31 ± 0.69	2.51 ± 1.57	0.67
Mast cell density (mean ± SEM)	30.00 ± 2.51	21.25 ± 3.32	0.34
Neutrophil/Eosinophil density (mean ± SEM)	155.74 ± 29.28	220.87 ± 94.11	0.39
Mature Dendritic cell density (mean ± SEM)	3.32 ± 0.32	3.46 ± 0.67	0.59
Immature Dendritic cell density (mean ± SEM)	118.52 ± 11.86	108.79 ± 19.72	0.89
$CD163^-$ Monocyte -Macrophage density (mean ± SEM)	51.68 ± 4.17	37.79 ± 5.50	0.30
$CD163^+$ Monocyte -Macrophage density (mean ± SEM)	73.94 ± 7.16	62.31 ± 14.26	0.59
$CD8^+/CD68^+$ Ratio (mean ± SEM)	1.86 ± 0.24	2.41 ± 0.89	0.21
Total $CD68^+$ cell density (mean ± SEM)	125.61 ± 9.84	100.09 ± 17.97	0.35

bioRxiv preprint doi: https://doi.org/10.1101/2023.10.20.563335; this version posted October 23, 2023. The copyright holder for this preprint (which was not certified by peer review) is the author/funder, who has granted bioRxiv a license to display the preprint in perpetuity. It is made available under aCC-BY 4.0 International license.

Supplementary Table 2. Table of antibodies used in the mIHC panel.

Primary Antibody	Clone	Species	Supplier	Catalog number
TOX1 (Tox)	NAN488B	Rat	Abcam	ab237009
PD-1 (PD1)	NAT105	Mouse	Abcam	ab52597
T-BET (TBET)	D6N8B	Rabbit	Cell Signaling Technologies	13232S
CD39	A1	Mouse	Biolegend	328202
TIM3	D5D5R	Rabbit	Cell Signaling Technologies	45208S
LAG-3 (Lag3)	17B4	Mouse	Novus Biologicals	NBP1-97657
TCF-1 (TCF1/7)	C6D39	Rabbit	Cell Signaling Technologies	2203
CD44	156-3C11	Mouse	Cell Signaling Technologies	3570
CD38	38C03	Mouse	Thermofisher Scientific	MA5-14413
CD3	SP7	Rabbit	Thermofisher Scientific	RM-9107-S
CD45RO	UCHL-1	Mouse	Thermofisher Scientific	MA5-11532
GrzB (Granzyme B)	Polyclonal	Rabbit	Abcam	ab4059
CD8	C8/144B	Mouse	Thermofisher Scientific	MA5-13473
CD11b (CD11B)	EPR1344	Rabbit	Abcam	ab133357
CD45RA	F8-11-13	Mouse	Abcam	ab59168
FOXP3 (Foxp3)	236A/E7	Mouse	eBioscience	14-4777-82
EOMES	Polyclonal	Rabbit	EMD Millipore	AB2283
CD20	L26	Mouse	Abcam	ab9475
Ki-67 (KI67)	SP6	Rabbit	Sigma/Cell Marque	275R-14
αSMA (Alpha-SMA)	Polyclonal	Rabbit	Abcam	ab5694
PanCK (Pan-Cytokeratin)	AE1/AE3	Mouse	Abcam	ab27988

bioRxiv preprint doi: https://doi.org/10.1101/2023.10.20.563335; this version posted October 23, 2023. The copyright holder for this preprint (which was not certified by peer review) is the author/funder, who has granted bioRxiv a license to display the preprint in perpetuity. It is made available under aCC-BY 4.0 International license.

Supplementary Table S3. Raw counts of cell states defined by mIHC gating strategy present in the dataset.

Cell State	Count
Ki-67- Neoplastic	915586
Myeloid cells	593571
Mesenchymal	247778
CD44- CD4 Th	163809
CD44- CD8 T_{OTHER}	136543
B cells	132728
Ki-67+ Neoplastic	115294
mT_{REG}	30836
CD44+ CD4 Th	24232
CD44+ CD8 T_{OTHER}	21711
CD44- CD4 $Th1_{OTHER}$	21157
Naive T_{REG}	10075
TBET+ T_{REG}	3674
CD8 T_{EMRA}	3550
CD8 T_{NAIVE}	2328
CD4 $Th1_{EM}$	2038
CD4 $Th1_{EMRA}$	1165
CD8 T_{EM}	940
CD8 T_{EX}	525
CD44+ CD4 $Th1_{OTHER}$	449
CD8 T_{TEX}	200
CD8 T_{EFF}	64
CD4 $Th1_{EFF}$	21

bioRxiv preprint doi: https://doi.org/10.1101/2023.10.20.563335; this version posted October 23, 2023. The copyright holder for this preprint (which was not certified by peer review) is the author/funder, who has granted bioRxiv a license to display the preprint in perpetuity. It is made available under aCC-BY 4.0 International license.

Supplementary Table S4. Raw counts of T cells expressing each functionality barcode present in the dataset.

T cell Functionality Barcode	Count
Negative for all	144053
TOX1+	51606
TCF-1+	50915
CD44+	19724
T-BET+	15539
EOMES+	15405
TCF-1+ T-BET+	12775
TOX1+ TCF-1+	10565
CD44+ TCF-1+	7742
EOMES+ TCF-1+	6118
TOX1+ T-BET+	5000
TOX1+ EOMES+	4876
TOX1+ TCF-1+ T-BET+	4403
EOMES+ CD44+	3505
EOMES+ CD44+ TCF-1+	3318
TOX1+ CD44+	3002
PD-1+ TOX1+ TCF-1+	2684
PD-1+ TOX1+	2669
CD44+ T-BET+	2174
CD44+ TCF-1+ T-BET+	2103
EOMES+ TCF-1+ T-BET+	1862
CD39+	1804
TOX1+ EOMES+ TCF-1+	1629
TOX1+ CD44+ TCF-1+	1591
TOX1+ TIM3+	1574
TOX1+ CD39+	1472
CD38+ CD44+	1356
PD-1+ TOX1+ TCF-1+ T-BET+	1262
PD-1+	1177
TOX1+ EOMES+ TCF-1+ T-BET+	1153
EOMES+ T-BET+	1152
TIM3+	1084
TOX1+ CD38+	949
CD38+	936
PD-1+ TOX1+ EOMES+ TCF-1+ T-BET+	923
TOX1+ EOMES+ CD44+	851
PD-1+ TOX1+ EOMES+	840
PD-1+ TCF-1+	833
LAG-3+	819
PD-1+ TOX1+ EOMES+ TCF-1+	785
EOMES+ CD44+ TCF-1+ T-BET+	751
PD-1+ TOX1+ TIM3+ LAG-3+ CD39+ EOMES+ CD38+ CD44+ TCF-1+ T-BET+	728
TOX1+ EOMES+ T-BET+	582

bioRxiv preprint doi: https://doi.org/10.1101/2023.10.20.563335; this version posted October 23, 2023. The copyright holder for this preprint (which was not certified by peer review) is the author/funder, who has granted bioRxiv a license to display the preprint in perpetuity. It is made available under aCC-BY 4.0 International license.

PD-1+ TOX1+ CD44+ TCF-1+	553
TOX1+ CD38+ CD44+	512
TOX1+ CD39+ EOMES+	495
TOX1+ CD44+ TCF-1+ T-BET+	483
CD39+ EOMES+	478
LAG-3+ TCF-1+	473
TOX11+ CD44+ T-BET+	461
PD-1+ TOX11+ T-BET+	457
CD39+ TCF-1+	448
TOX11+ EOMES+ CD44+ TCF-1+	437
TIM3+ CD44+	430
PD-1+ T-BET+	386
EOMES+ CD38+ CD44+	351
CD38+ CD44+ TCF-1+	341
EOMES+ CD38+ CD44+ TCF-1+	319
PD-1+ TOX1+ CD39+	310
TOX1+ LAG-3+ CD39+	304
TOX1+ LAG-3+	303
EOMES+ CD44+ T-BET+	292
TIM3+ TCF-1+	281
LAG-3+ T-BET+	269
PD-1+ EOMES+ TCF-1+	256
EOMES+ CD38+	252
TIM3+ T-BET+	249
PD-1+ TOX1+ TIM3+ LAG-3+ CD39+ EOMES+ CD38+ CD44+ TCF-1+	247
PD-1+ TOX1+ CD44+	244
CD38+ TCF-1+	228
PD-1+ TCF-1+ T-BET+	216
PD-1+ EOMES+	216
TOX1+ TIM3+ T-BET+	208
LAG-3+ EOMES+ TCF-1+	198
LAG-3+ TCF-1+ T-BET+	193
CD39+ CD44+	190
TOX1+ CD38+ CD44+ TCF-1+	178
PD-1+ TOX1+ LAG-3+ CD39+	175
PD-1+ TOX1+ CD44+ TCF-1+ T-BET+	173
LAG-3+ EOMES+	167
PD-1+ CD44+ TCF-1+	166
TOX1+ TIM3+ CD39+ CD44+ TCF-1+ T-BET+	165
TOX1+ EOMES+ CD44+ TCF-1+ T-BET+	162
CD39+ T-BET+	161
TOX1+ TIM3+ CD44+	157
TOX1+ TIM3+ TCF-1+	150
PD-1+ CD44+	149

bioRxiv preprint doi: https://doi.org/10.1101/2023.10.20.563335; this version posted October 23, 2023. The copyright holder for this preprint (which was not certified by peer review) is the author/funder, who has granted bioRxiv a license to display the preprint in perpetuity. It is made available under aCC-BY 4.0 International license.

TIM3+ TCF-1+ T-BET+	144
TOX1+ EOMES+ CD44+ T-BET+	144
CD39+ EOMES+ TCF-1+	144
CD39+ TCF-1+ T-BET+	141
PD-1+ TOX1+ EOMES+ T-BET+	139
TOX1+ CD39+ EOMES+ TCF-1+	138
TOX1+ TIM3+ CD39+ CD38+ CD44+ TCF-1+ T-BET+	137
PD-1+ TOX1+ LAG-3+ CD39+ EOMES+	135
TOX1+ EOMES+ CD38+ CD44+ TCF-1+	133
TOX1+ CD39+ TCF-1+	133
PD-1+ TOX1+ TIM3+ LAG-3+ CD39+ CD38+ CD44+ TCF-1+ T-BET+	129
TOX1+ CD38+ T-BET+	125
PD-1+ TOX1+ CD39+ EOMES+	124
EOMES+ CD38+ TCF-1+	124
TOX1+ LAG-3+ TCF-1+	122
PD-1+ TOX1+ LAG-3+	118
PD-1+ TOX1+ EOMES+ CD44+ TCF-1+	116
TOX1+ LAG-3+ EOMES+	115
PD-1+ EOMES+ TCF-1+ T-BET+	115
TIM3+ EOMES+	113
PD-1+ TOX1+ LAG-3+ CD39+ EOMES+ CD38+ CD44+ TCF-1+	112
TOX1+ EOMES+ CD38+ CD44+	111
PD-1+ TOX1+ TIM3+	109
LAG-3+ CD44+ TCF-1+	109
LAG-3+ CD39+	107
LAG-3+ CD44+	107
TOX1+ TIM3+ EOMES+	105
TIM3+ CD44+ TCF-1+	103
PD-1+ TOX1+ CD39+ EOMES+ TCF-1+	101
CD38+ T-BET+	100
TOX1+ CD39+ EOMES+ CD38+ TCF-1+	99
TIM3+ CD39+	97
TOX1+ TIM3+ TCF-1+ T-BET+	97
PD-1+ TOX1+ EOMES+ CD44+ TCF-1+ T-BET+	97
LAG-3+ EOMES+ TCF-1+ T-BET+	95
CD39+ EOMES+ TCF-1+ T-BET+	94
CD39+ CD38+	94
TOX1+ LAG-3+ CD39+ EOMES+	94
PD-1+ TOX1+ TIM3+ LAG-3+ EOMES+ CD38+ CD44+ TCF-1+ T-BET+	94
CD38+ CD44+ T-BET+	93
CD39+ EOMES+ CD38+ CD44+	93
TOX1+ CD39+ T-BET+	91
TIM3+ CD44+ T-BET+	91
CD38+ TCF-1+ T-BET+	90

bioRxiv preprint doi: https://doi.org/10.1101/2023.10.20.563335; this version posted October 23, 2023. The copyright holder for this preprint (which was not certified by peer review) is the author/funder, who has granted bioRxiv a license to display the preprint in perpetuity. It is made available under aCC-BY 4.0 International license.

CD39+ EOMES+ CD38+	90
TOX1+ EOMES+ CD38+	88
TOX1+ CD38+ TCF-1+	88
TOX1+ CD39+ EOMES+ CD44+ TCF-1+ T-BET+	88
TOX1+ LAG-3+ EOMES+ TCF-1+	86
CD38+ CD44+ TCF-1+ T-BET+	85
LAG-3+ EOMES+ CD44+ TCF-1+	83
CD39+ CD38+ CD44+	83
TOX1+ TIM3+ CD39+ EOMES+ CD38+ CD44+ TCF-1+ T-BET+	82
TOX1+ TIM3+ LAG-3+ CD39+ EOMES+ CD38+ CD44+ TCF-1+ T-BET+	78
PD-1+ TOX1+ LAG-3+ EOMES+	75
TOX1+ EOMES+ CD38+ TCF-1+	75
PD-1+ TOX1+ CD39+ TCF-1+	73
PD-1+ CD44+ TCF-1+ T-BET+	71
PD-1+ TOX1+ CD38+	70
PD-1+ EOMES+ T-BET+	69
PD-1+ TOX1+ TIM3+ LAG-3+ CD38+ CD44+ TCF-1+ T-BET+	67
PD-1+ TOX1+ EOMES+ CD44+	67
PD-1+ EOMES+ CD44+ TCF-1+ T-BET+	67
TOX1+ CD39+ EOMES+ CD38+ CD44+ TCF-1+	64
TOX1+ CD39+ TCF-1+ T-BET+	63
PD-1+ TOX1+ TIM3+ LAG-3+ CD39+ EOMES+ CD44+ TCF-1+ T-BET+	63
TIM3+ EOMES+ CD44+	62
CD39+ EOMES+ T-BET+	61
TOX1+ TIM3+ EOMES+ TCF-1+	61
TIM3+ EOMES+ T-BET+	60
TOX1+ LAG-3+ T-BET+	60
PD-1+ EOMES+ CD44+ TCF-1+	59
CD39+ EOMES+ CD44+	59
PD-1+ TOX1+ LAG-3+ EOMES+ TCF-1+	59
LAG-3+ CD39+ TCF-1+	57
PD-1+ TOX1+ CD44+ T-BET+	57
PD-1+ TOX1+ LAG-3+ CD39+ EOMES+ CD38+ TCF-1+	56
LAG-3+ CD44+ TCF-1+ T-BET+	56
TIM3+ EOMES+ TCF-1+	54
PD-1+ TOX1+ LAG-3+ T-BET+	53
PD-1+ TOX1+ CD39+ EOMES+ TCF-1+ T-BET+	53
CD39+ EOMES+ CD38+ CD44+ TCF-1+	53
PD-1+ TOX1+ TIM3+ TCF-1+	53
CD39+ CD44+ TCF-1+	52
TOX1+ TIM3+ CD44+ TCF-1+	52
PD-1+ TOX1+ LAG-3+ TCF-1+	51
TOX1+ CD39+ EOMES+ CD38+	50
CD39+ EOMES+ CD38+ TCF-1+	49

bioRxiv preprint doi: https://doi.org/10.1101/2023.10.20.563335; this version posted October 23, 2023. The copyright holder for this preprint (which was not certified by peer review) is the author/funder, who has granted bioRxiv a license to display the preprint in perpetuity. It is made available under aCC-BY 4.0 International license.

PD-1+ CD44+ T-BET+	48
TOX1+ CD39+ EOMES+ CD38+ CD44+	47
TIM3+ EOMES+ CD44+ TCF-1+	47
PD-1+ TOX1+ CD39+ TCF-1+ T-BET+	47
TOX1+ CD39+ CD44+ TCF-1+ T-BET+	47
CD39+ EOMES+ CD44+ TCF-1+	46
PD-1+ TIM3+ LAG-3+ CD39+ EOMES+ CD38+ CD44+ TCF-1+ T-BET+	45
PD-1+ TOX1+ TIM3+ TCF-1+ T-BET+	44
PD-1+ TOX1+ TIM3+ LAG-3+ EOMES+ CD38+ CD44+ TCF-1+	43
TOX1+ CD39+ CD44+	43
CD39+ EOMES+ CD44+ TCF-1+ T-BET+	43
LAG-3+ CD38+ CD44+ TCF-1+	43
TOX1+ TIM3+ CD39+	43
LAG-3+ EOMES+ CD44+	42
PD-1+ TOX1+ LAG-3+ CD39+ EOMES+ CD38+ CD44+	42
TOX1+ CD38+ CD44+ TCF-1+ T-BET+	42
EOMES+ CD38+ CD44+ TCF-1+ T-BET+	42
LAG-3+ EOMES+ T-BET+	41
TIM3+ CD39+ TCF-1+	41
PD-1+ TOX1+ LAG-3+ CD39+ EOMES+ CD38+	41
PD-1+ CD39+	40
LAG-3+ EOMES+ CD38+ CD44+ TCF-1+	40
PD-1+ TOX1+ LAG-3+ CD39+ EOMES+ CD38+ CD44+ TCF-1+ T-BET+	40
LAG-3+ CD39+ EOMES+	39
PD-1+ EOMES+ CD44+	39
PD-1+ TOX1+ TIM3+ LAG-3+ CD39+ EOMES+ CD38+ TCF-1+	38
PD-1+ TOX1+ TIM3+ CD39+ EOMES+ CD38+ CD44+ TCF-1+ T-BET+	37
PD-1+ TOX1+ LAG-3+ EOMES+ T-BET+	37
TOX1+ LAG-3+ TCF-1+ T-BET+	37
PD-1+ TOX1+ TIM3+ T-BET+	37
TOX1+ TIM3+ CD38+ CD44+	37
PD-1+ TOX1+ CD38+ CD44+	37
PD-1+ TOX1+ LAG-3+ CD39+ EOMES+ TCF-1+	36
TOX1+ TIM3+ LAG-3+ EOMES+ CD38+ CD44+ TCF-1+ T-BET+	36
TOX1+ CD39+ EOMES+ T-BET+	35
TOX1+ TIM3+ EOMES+ CD44+	35
PD-1+ TOX1+ TIM3+ CD38+ CD44+ TCF-1+ T-BET+	34
TIM3+ CD39+ CD44+	34
LAG-3+ EOMES+ CD44+ TCF-1+ T-BET+	34
TOX1+ TIM3+ CD38+	34
TOX1+ CD38+ TCF-1+ T-BET+	33
PD-1+ LAG-3+ EOMES+ TCF-1+	33
PD-1+ TOX1+ TIM3+ CD39+ EOMES+ CD38+ CD44+ TCF-1+	32
PD-1+ TOX1+ TIM3+ CD38+ CD44+ T-BET+	32

bioRxiv preprint doi: https://doi.org/10.1101/2023.10.20.563335; this version posted October 23, 2023. The copyright holder for this preprint (which was not certified by peer review) is the author/funder, who has granted bioRxiv a license to display the preprint in perpetuity. It is made available under aCC-BY 4.0 International license.

LAG-3+ CD44+ T-BET+	32
TOX1+ CD39+ EOMES+ CD44+ TCF-1+	32
TIM3+ CD44+ TCF-1+ T-BET+	32
TIM3+ CD39+ CD38+	32
CD39+ CD44+ TCF-1+ T-BET+	32
TIM3+ CD38+ CD44+	31
LAG-3+ EOMES+ CD38+ CD44+	31
PD-1+ TOX1+ TIM3+ LAG-3+ CD39+ CD44+ TCF-1+ T-BET+	31
PD-1+ LAG-3+ EOMES+	31
PD-1+ TOX1+ EOMES+ CD44+ T-BET+	31
TOX1+ TIM3+ LAG-3+ CD39+ CD38+ CD44+ TCF-1+ T-BET+	31
LAG-3+ CD38+ TCF-1+	31
PD-1+ TOX1+ TIM3+ LAG-3+ EOMES+ CD38+ TCF-1+ T-BET+	30
TOX1+ CD39+ CD38+	30
TOX1+ TIM3+ CD44+ T-BET+	30
TIM3+ EOMES+ TCF-1+ T-BET+	30
TOX1+ LAG-3+ CD39+ EOMES+ CD44+ TCF-1+ T-BET+	30
PD-1+ LAG-3+	30
PD-1+ TOX1+ LAG-3+ CD39+ EOMES+ TCF-1+ T-BET+	30
TOX1+ CD39+ CD44+ TCF-1+	29
PD-1+ TOX1+ CD39+ T-BET+	29
TOX1+ TIM3+ CD44+ TCF-1+ T-BET+	29
PD-1+ TOX1+ TIM3+ LAG-3+ CD39+ EOMES+ CD38+ CD44+ T-BET+	29
LAG-3+ CD39+ EOMES+ CD38+ CD44+ TCF-1+	29
LAG-3+ CD39+ EOMES+ CD38+ CD44+	29
TIM3+ LAG-3+	29
LAG-3+ CD38+	28
TOX1+ EOMES+ CD38+ CD44+ TCF-1+ T-BET+	28
LAG-3+ CD39+ EOMES+ TCF-1+ T-BET+	28
TIM3+ LAG-3+ T-BET+	28
TOX1+ LAG-3+ EOMES+ TCF-1+ T-BET+	28
TOX1+ CD39+ EOMES+ TCF-1+ T-BET+	28
TOX1+ TIM3+ EOMES+ CD44+ TCF-1+	28
LAG-3+ CD39+ EOMES+ CD38+	28
PD-1+ TOX1+ LAG-3+ EOMES+ CD38+ TCF-1+	27
TIM3+ EOMES+ CD44+ TCF-1+ T-BET+	27
PD-1+ TOX1+ LAG-3+ CD39+ TCF-1+	27
PD-1+ LAG-3+ TCF-1+	27
PD-1+ LAG-3+ CD38+ TCF-1+ T-BET+	27
TOX1+ LAG-3+ CD39+ TCF-1+	27
PD-1+ TOX1+ LAG-3+ CD39+ T-BET+	27
TOX1+ CD38+ CD44+ T-BET+	26
TOX1+ TIM3+ LAG-3+ CD39+ EOMES+ CD38+ CD44+ TCF-1+	26
PD-1+ TOX1+ LAG-3+ EOMES+ CD38+	26

bioRxiv preprint doi: https://doi.org/10.1101/2023.10.20.563335; this version posted October 23, 2023. The copyright holder for this preprint (which was not certified by peer review) is the author/funder, who has granted bioRxiv a license to display the preprint in perpetuity. It is made available under aCC-BY 4.0 International license.

LAG-3+ EOMES+ CD38+ TCF-1+	26
TOX1+ LAG-3+ CD39+ EOMES+ CD38+ TCF-1+	26
LAG-3+ CD39+ EOMES+ CD38+ TCF-1+	26
TOX1+ TIM3+ LAG-3+ CD38+ CD44+ TCF-1+ T-BET+	26
PD-1+ TOX1+ TIM3+ CD39+ EOMES+ CD44+ TCF-1+	26
LAG-3+ CD39+ EOMES+ TCF-1+	26
PD-1+ TIM3+	25
CD39+ CD38+ CD44+ TCF-1+	25
PD-1+ TOX1+ LAG-3+ TCF-1+ T-BET+	25
PD-1+ TIM3+ LAG-3+ EOMES+ CD38+ CD44+ TCF-1+ T-BET+	25
LAG-3+ CD38+ CD44+	25
TOX1+ TIM3+ LAG-3+	25
LAG-3+ CD39+ TCF-1+ T-BET+	25
TOX1+ LAG-3+ EOMES+ T-BET+	25
TOX1+ TIM3+ CD38+ CD44+ TCF-1+ T-BET+	25
TOX1+ TIM3+ LAG-3+ EOMES+ CD38+ TCF-1+ T-BET+	24
PD-1+ TOX1+ CD39+ EOMES+ CD38+	24
CD39+ CD38+ TCF-1+	24
TIM3+ EOMES+ CD38+ CD44+ TCF-1+	24
TOX1+ TIM3+ EOMES+ CD38+ CD44+ TCF-1+	23
PD-1+ TOX1+ TIM3+ CD44+ TCF-1+	23
TOX1+ LAG-3+ CD39+ EOMES+ CD38+	23
PD-1+ TOX1+ CD39+ CD44+	23
PD-1+ TOX1+ CD39+ EOMES+ CD38+ TCF-1+	23
PD-1+ TOX1+ TIM3+ EOMES+ CD38+ CD44+ TCF-1+ T-BET+	23
CD39+ CD44+ T-BET+	23
PD-1+ TOX1+ CD39+ EOMES+ CD44+ TCF-1+	23
TIM3+ LAG-3+ CD39+ EOMES+ CD38+ CD44+ TCF-1+ T-BET+	22
LAG-3+ CD38+ CD44+ TCF-1+ T-BET+	22
TOX1+ TIM3+ EOMES+ T-BET+	22
TOX1+ CD39+ EOMES+ CD44+	22
PD-1+ TOX1+ LAG-3+ CD39+ EOMES+ CD38+ TCF-1+ T-BET+	22
EOMES+ CD38+ TCF-1+ T-BET+	22
LAG-3+ CD38+ TCF-1+ T-BET+	22
TIM3+ CD39+ T-BET+	22
TOX1+ LAG-3+ CD39+ T-BET+	22
LAG-3+ CD39+ EOMES+ CD44+ TCF-1+ T-BET+	22
PD-1+ TOX1+ LAG-3+ EOMES+ CD38+ CD44+ TCF-1+	22
PD-1+ LAG-3+ EOMES+ CD44+ TCF-1+	21
PD-1+ TOX1+ CD39+ CD44+ TCF-1+	21
PD-1+ EOMES+ CD44+ T-BET+	21
TIM3+ LAG-3+ CD38+ CD44+ TCF-1+ T-BET+	21
TIM3+ LAG-3+ EOMES+ TCF-1+	21
PD-1+ TOX1+ LAG-3+ EOMES+ CD38+ CD44+	21

bioRxiv preprint doi: https://doi.org/10.1101/2023.10.20.563335; this version posted October 23, 2023. The copyright holder for this preprint (which was not certified by peer review) is the author/funder, who has granted bioRxiv a license to display the preprint in perpetuity. It is made available under aCC-BY 4.0 International license.

TOX1+ TIM3+ EOMES+ TCF-1+ T-BET+	21
PD-1+ TOX1+ LAG-3+ CD39+ EOMES+ T-BET+	21
TOX1+ EOMES+ CD38+ T-BET+	21
TOX1+ LAG-3+ EOMES+ CD38+ TCF-1+	20
TIM3+ LAG-3+ TCF-1+	20
TOX1+ LAG-3+ CD39+ EOMES+ CD38+ CD44+	20
TOX1+ TIM3+ CD38+ TCF-1+ T-BET+	20
TOX1+ TIM3+ EOMES+ CD38+ CD44+ TCF-1+ T-BET+	20
PD-1+ TOX1+ TIM3+ CD44+	20
TOX1+ TIM3+ CD39+ TCF-1+	20
PD-1+ TOX1+ EOMES+ CD38+	20
EOMES+ CD38+ CD44+ T-BET+	20
PD-1+ TOX1+ CD38+ CD44+ TCF-1+	20
PD-1+ TOX1+ CD39+ EOMES+ T-BET+	19
TOX1+ CD39+ CD44+ T-BET+	19
TIM3+ LAG-3+ EOMES+ CD38+ CD44+ TCF-1+	19
LAG-3+ CD39+ T-BET+	19
TOX1+ TIM3+ LAG-3+ EOMES+ CD38+ TCF-1+	19
LAG-3+ EOMES+ CD38+	19
PD-1+ TOX1+ TIM3+ LAG-3+ CD39+ CD38+ CD44+ T-BET+	19
TOX1+ TIM3+ LAG-3+ EOMES+ CD38+ CD44+ TCF-1+	19
PD-1+ CD39+ EOMES+	18
CD39+ CD38+ CD44+ TCF-1+ T-BET+	18
TIM3+ CD39+ CD38+ CD44+	18
TIM3+ LAG-3+ EOMES+ CD38+ CD44+ TCF-1+ T-BET+	18
PD-1+ TOX1+ CD38+ TCF-1+	18
PD-1+ TOX1+ LAG-3+ EOMES+ TCF-1+ T-BET+	18
TIM3+ CD38+	18
TOX1+ LAG-3+ CD44+ TCF-1+	18
LAG-3+ CD39+ CD44+ TCF-1+	17
PD-1+ CD39+ TCF-1+	17
CD39+ CD38+ T-BET+	17
PD-1+ TOX1+ TIM3+ EOMES+ TCF-1+	17
TIM3+ CD38+ CD44+ TCF-1+ T-BET+	17
PD-1+ TOX1+ CD38+ T-BET+	17
PD-1+ TOX1+ EOMES+ CD38+ CD44+	17
PD-1+ TOX1+ CD39+ EOMES+ CD38+ CD44+	17
PD-1+ TOX1+ TIM3+ EOMES+	17
PD-1+ TOX1+ TIM3+ LAG-3+ CD39+ EOMES+ CD44+ TCF-1+	17
PD-1+ TOX1+ TIM3+ CD39+ CD38+ CD44+ TCF-1+ T-BET+	17
PD-1+ TOX1+ TIM3+ LAG-3+ CD44+ TCF-1+ T-BET+	17
TIM3+ EOMES+ CD44+ T-BET+	17
TOX1+ LAG-3+ CD39+ EOMES+ CD38+ TCF-1+ T-BET+	17
LAG-3+ CD39+ CD38+	17

bioRxiv preprint doi: https://doi.org/10.1101/2023.10.20.563335; this version posted October 23, 2023. The copyright holder for this preprint (which was not certified by peer review) is the author/funder, who has granted bioRxiv a license to display the preprint in perpetuity. It is made available under aCC-BY 4.0 International license.

PD-1+ TOX1+ TIM3+ CD38+ CD44+	16
PD-1+ TOX1+ TIM3+ LAG-3+ CD39+ EOMES+ CD38+ TCF-1+ T-BET+	16
LAG-3+ EOMES+ CD38+ TCF-1+ T-BET+	16
PD-1+ LAG-3+ T-BET+	16
CD39+ CD38+ CD44+ T-BET+	16
PD-1+ LAG-3+ TCF-1+ T-BET+	16
TOX1+ LAG-3+ EOMES+ CD38+	16
TOX1+ TIM3+ EOMES+ CD38+ TCF-1+	16
TOX1+ LAG-3+ CD39+ EOMES+ TCF-1+ T-BET+	16
PD-1+ TOX1+ LAG-3+ EOMES+ CD44+	15
TOX1+ LAG-3+ CD39+ EOMES+ CD44+ TCF-1+	15
PD-1+ TOX1+ LAG-3+ CD44+	15
PD-1+ LAG-3+ CD38+ TCF-1+	15
PD-1+ TOX1+ CD39+ EOMES+ CD44+ TCF-1+ T-BET+	14
PD-1+ TOX1+ EOMES+ CD38+ CD44+ TCF-1+	14
LAG-3+ CD39+ CD38+ CD44+ TCF-1+	14
LAG-3+ EOMES+ CD38+ CD44+ TCF-1+ T-BET+	14
LAG-3+ CD39+ CD38+ T-BET+	14
EOMES+ CD38+ T-BET+	14
TOX1+ LAG-3+ CD44+	14
PD-1+ TOX1+ LAG-3+ CD39+ CD44+ TCF-1+ T-BET+	14
TOX1+ TIM3+ EOMES+ CD44+ TCF-1+ T-BET+	14
PD-1+ TOX1+ TIM3+ CD39+ CD38+ CD44+ T-BET+	14
PD-1+ LAG-3+ CD39+	14
PD-1+ TOX1+ EOMES+ CD38+ CD44+ TCF-1+ T-BET+	14
PD-1+ LAG-3+ CD39+ T-BET+	14
TOX1+ LAG-3+ EOMES+ CD38+ CD44+ TCF-1+	14
TOX1+ EOMES+ CD38+ TCF-1+ T-BET+	14
TIM3+ LAG-3+ EOMES+ T-BET+	14
TIM3+ CD39+ EOMES+	14
PD-1+ TIM3+ T-BET+	14
PD-1+ CD39+ TCF-1+ T-BET+	14
PD-1+ TOX1+ LAG-3+ CD39+ TCF-1+ T-BET+	13
TOX1+ LAG-3+ CD38+ TCF-1+ T-BET+	13
PD-1+ TOX1+ CD38+ TCF-1+ T-BET+	13
TIM3+ LAG-3+ CD38+ TCF-1+ T-BET+	13
LAG-3+ CD39+ CD38+ TCF-1+ T-BET+	13
LAG-3+ CD39+ EOMES+ CD38+ TCF-1+ T-BET+	13
TOX1+ LAG-3+ EOMES+ CD38+ TCF-1+ T-BET+	13
TOX1+ LAG-3+ CD39+ EOMES+ CD38+ CD44+ TCF-1+	13
PD-1+ TOX1+ CD39+ EOMES+ CD44+	13
PD-1+ TIM3+ LAG-3+ CD38+ TCF-1+ T-BET+	13
PD-1+ TOX1+ TIM3+ CD44+ TCF-1+ T-BET+	13
TOX1+ CD39+ CD38+ CD44+	13

bioRxiv preprint doi: https://doi.org/10.1101/2023.10.20.563335; this version posted October 23, 2023. The copyright holder for this preprint (which was not certified by peer review) is the author/funder, who has granted bioRxiv a license to display the preprint in perpetuity. It is made available under aCC-BY 4.0 International license.

TOX1+ LAG-3+ CD39+ CD38+	13
TOX1+ TIM3+ CD39+ EOMES+	13
TOX1+ CD39+ CD38+ T-BET+	13
PD-1+ TOX1+ TIM3+ CD38+ TCF-1+ T-BET+	13
PD-1+ TOX1+ TIM3+ EOMES+ TCF-1+ T-BET+	13
LAG-3+ CD39+ CD38+ CD44+	12
TIM3+ CD39+ TCF-1+ T-BET+	12
PD-1+ TOX1+ LAG-3+ EOMES+ CD44+ TCF-1+	12
PD-1+ TOX1+ LAG-3+ CD39+ CD38+ TCF-1+	12
CD39+ CD38+ TCF-1+ T-BET+	12
PD-1+ TOX1+ LAG-3+ CD44+ TCF-1+	12
PD-1+ TOX1+ LAG-3+ CD44+ TCF-1+ T-BET+	12
PD-1+ TOX1+ CD39+ CD38+	12
PD-1+ TOX1+ EOMES+ CD38+ TCF-1+	12
TOX1+ LAG-3+ EOMES+ CD44+ TCF-1+	12
PD-1+ TOX1+ TIM3+ LAG-3+ EOMES+ TCF-1+	12
PD-1+ TIM3+ TCF-1+	12
TOX1+ LAG-3+ CD39+ CD38+ TCF-1+ T-BET+	12
TOX1+ TIM3+ EOMES+ CD38+ TCF-1+ T-BET+	12
PD-1+ LAG-3+ CD39+ EOMES+ TCF-1+	12
LAG-3+ EOMES+ CD44+ T-BET+	12
PD-1+ TIM3+ CD44+	12
TOX1+ LAG-3+ CD38+	12
TOX1+ LAG-3+ CD39+ EOMES+ CD38+ CD44+ TCF-1+ T-BET+	12
TOX1+ LAG-3+ CD44+ T-BET+	12
TIM3+ LAG-3+ CD39+	12
TIM3+ LAG-3+ EOMES+ CD38+ TCF-1+ T-BET+	12
TOX1+ LAG-3+ EOMES+ CD38+ CD44+	12
LAG-3+ CD38+ T-BET+	12
CD39+ EOMES+ CD38+ TCF-1+ T-BET+	12
TIM3+ LAG-3+ CD38+ T-BET+	12
PD-1+ CD39+ EOMES+ TCF-1+ T-BET+	12
TIM3+ LAG-3+ CD39+ EOMES+ CD44+ TCF-1+	12
PD-1+ CD39+ T-BET+	12
PD-1+ TOX1+ LAG-3+ CD39+ EOMES+ CD44+ TCF-1+	12
PD-1+ TOX1+ EOMES+ CD38+ TCF-1+ T-BET+	11
PD-1+ LAG-3+ EOMES+ CD44+ TCF-1+ T-BET+	11
PD-1+ LAG-3+ EOMES+ TCF-1+ T-BET+	11
LAG-3+ CD39+ EOMES+ CD38+ CD44+ TCF-1+ T-BET+	11
TOX1+ LAG-3+ CD39+ TCF-1+ T-BET+	11
TOX1+ CD39+ EOMES+ CD44+ T-BET+	11
PD-1+ LAG-3+ CD44+ TCF-1+	11
CD39+ EOMES+ CD38+ CD44+ TCF-1+ T-BET+	11
PD-1+ TOX1+ TIM3+ LAG-3+ CD38+ TCF-1+ T-BET+	11

bioRxiv preprint doi: https://doi.org/10.1101/2023.10.20.563335; this version posted October 23, 2023. The copyright holder for this preprint (which was not certified by peer review) is the author/funder, who has granted bioRxiv a license to display the preprint in perpetuity. It is made available under aCC-BY 4.0 International license.

TIM3+ CD38+ T-BET+	11
TOX1+ TIM3+ EOMES+ CD38+ CD44+	11
TIM3+ LAG-3+ EOMES+	11
TOX1+ TIM3+ EOMES+ CD44+ T-BET+	11
TOX1+ TIM3+ LAG-3+ CD39+ EOMES+ CD44+ TCF-1+ T-BET+	11
PD-1+ TOX1+ LAG-3+ CD39+ CD44+ TCF-1+	11
TIM3+ LAG-3+ CD44+	11
LAG-3+ CD39+ EOMES+ CD44+ TCF-1+	11
LAG-3+ CD39+ EOMES+ CD44+	11
PD-1+ TOX1+ TIM3+ CD38+	11
TOX1+ TIM3+ CD38+ T-BET+	11
PD-1+ TOX1+ LAG-3+ EOMES+ CD38+ CD44+ TCF-1+ T-BET+	11
PD-1+ CD38+	11
TOX1+ TIM3+ CD38+ TCF-1+	11
TIM3+ CD38+ TCF-1+ T-BET+	10
PD-1+ TOX1+ TIM3+ LAG-3+ EOMES+ CD44+ TCF-1+ T-BET+	10
PD-1+ TOX1+ LAG-3+ EOMES+ CD38+ CD44+ T-BET+	10
PD-1+ LAG-3+ CD39+ EOMES+ CD38+ CD44+ TCF-1+ T-BET+	10
TIM3+ CD38+ CD44+ T-BET+	10
TIM3+ LAG-3+ CD38+ CD44+ T-BET+	10
PD-1+ TIM3+ LAG-3+ CD39+ CD38+ CD44+ T-BET+	10
TIM3+ LAG-3+ EOMES+ TCF-1+ T-BET+	10
PD-1+ TOX1+ CD39+ CD38+ CD44+	10
PD-1+ LAG-3+ EOMES+ T-BET+	10
PD-1+ TOX1+ CD39+ EOMES+ CD44+ T-BET+	10
PD-1+ TOX1+ TIM3+ CD39+	10
PD-1+ TOX1+ CD39+ EOMES+ CD38+ CD44+ TCF-1+	10
PD-1+ TOX1+ TIM3+ LAG-3+	10
PD-1+ TOX1+ TIM3+ LAG-3+ CD38+ CD44+ T-BET+	10
TOX1+ LAG-3+ CD39+ EOMES+ T-BET+	10
LAG-3+ CD39+ CD44+	10
TIM3+ CD39+ CD44+ TCF-1+	10
TOX1+ TIM3+ LAG-3+ EOMES+ TCF-1+	10
TIM3+ EOMES+ CD38+ CD44+ TCF-1+ T-BET+	9
PD-1+ TIM3+ EOMES+ CD38+ CD44+ TCF-1+ T-BET+	9
TIM3+ CD39+ CD38+ TCF-1+	9
PD-1+ CD39+ CD44+ TCF-1+	9
PD-1+ CD38+ TCF-1+	9
PD-1+ LAG-3+ CD38+ CD44+ TCF-1+ T-BET+	9
PD-1+ TIM3+ CD44+ TCF-1+ T-BET+	9
TOX1+ EOMES+ CD38+ CD44+ T-BET+	9
PD-1+ TOX1+ LAG-3+ EOMES+ CD44+ TCF-1+ T-BET+	9
PD-1+ TOX1+ TIM3+ CD39+ TCF-1+ T-BET+	9
LAG-3+ CD39+ CD38+ CD44+ TCF-1+ T-BET+	9

bioRxiv preprint doi: https://doi.org/10.1101/2023.10.20.563335; this version posted October 23, 2023. The copyright holder for this preprint (which was not certified by peer review) is the author/funder, who has granted bioRxiv a license to display the preprint in perpetuity. It is made available under aCC-BY 4.0 International license.

PD-1+ TOX1+ TIM3+ CD39+ EOMES+ TCF-1+	9
TOX1+ TIM3+ CD39+ EOMES+ TCF-1+	9
TOX1+ LAG-3+ CD38+ T-BET+	9
PD-1+ TOX1+ LAG-3+ CD38+	9
PD-1+ TOX1+ TIM3+ LAG-3+ EOMES+	9
TOX1+ TIM3+ CD39+ CD38+ TCF-1+ T-BET+	9
TOX1+ TIM3+ CD38+ CD44+ TCF-1+	9
PD-1+ TOX1+ TIM3+ LAG-3+ CD39+ EOMES+ CD38+	9
TOX1+ LAG-3+ CD39+ CD44+ TCF-1+	9
TIM3+ CD38+ CD44+ TCF-1+	9
PD-1+ LAG-3+ EOMES+ CD38+ CD44+	8
TOX1+ LAG-3+ EOMES+ CD44+	8
PD-1+ TOX1+ TIM3+ CD39+ CD44+ TCF-1+ T-BET+	8
PD-1+ TOX1+ TIM3+ EOMES+ CD44+ TCF-1+	8
PD-1+ LAG-3+ CD39+ EOMES+ TCF-1+ T-BET+	8
PD-1+ TOX1+ TIM3+ EOMES+ CD44+	8
PD-1+ TOX1+ TIM3+ CD39+ EOMES+	8
PD-1+ LAG-3+ CD39+ CD38+ TCF-1+ T-BET+	8
PD-1+ TOX1+ TIM3+ CD44+ T-BET+	8
LAG-3+ EOMES+ CD38+ T-BET+	8
PD-1+ TOX1+ TIM3+ LAG-3+ CD38+ CD44+	8
PD-1+ TOX1+ TIM3+ LAG-3+ CD39+ CD44+ TCF-1+	8
CD39+ EOMES+ CD38+ CD44+ T-BET+	8
PD-1+ TOX1+ TIM3+ LAG-3+ CD39+ EOMES+ TCF-1+	8
CD39+ EOMES+ CD38+ T-BET+	8
CD39+ EOMES+ CD44+ T-BET+	8
PD-1+ CD39+ EOMES+ CD44+ TCF-1+	8
TOX1+ CD39+ CD38+ TCF-1+	8
PD-1+ CD39+ EOMES+ CD44+	8
PD-1+ TOX1+ TIM3+ LAG-3+ CD39+ EOMES+ CD38+ CD44+	8
TOX1+ LAG-3+ CD39+ CD44+ TCF-1+ T-BET+	8
PD-1+ CD39+ EOMES+ TCF-1+	8
TOX1+ TIM3+ LAG-3+ TCF-1+	8
PD-1+ TOX1+ CD39+ CD44+ T-BET+	8
PD-1+ TOX1+ CD39+ EOMES+ CD38+ CD44+ T-BET+	8
TOX1+ LAG-3+ CD39+ EOMES+ CD44+	8
PD-1+ TOX1+ LAG-3+ EOMES+ CD44+ T-BET+	8
PD-1+ TOX1+ CD39+ CD44+ TCF-1+ T-BET+	8
TOX1+ TIM3+ LAG-3+ CD39+ EOMES+ CD38+ TCF-1+ T-BET+	8
PD-1+ TOX1+ CD38+ CD44+ TCF-1+ T-BET+	8
PD-1+ TOX1+ LAG-3+ CD39+ CD38+ CD44+ TCF-1+ T-BET+	8
TIM3+ EOMES+ CD38+ CD44+	8
TIM3+ LAG-3+ EOMES+ CD44+ TCF-1+ T-BET+	8
PD-1+ TIM3+ EOMES+	8

bioRxiv preprint doi: https://doi.org/10.1101/2023.10.20.563335; this version posted October 23, 2023. The copyright holder for this preprint (which was not certified by peer review) is the author/funder, who has granted bioRxiv a license to display the preprint in perpetuity. It is made available under aCC-BY 4.0 International license.

TOX1+ TIM3+ LAG-3+ CD39+	8
TOX1+ TIM3+ LAG-3+ CD39+ CD38+ TCF-1+ T-BET+	8
PD-1+ TOX1+ CD39+ EOMES+ CD38+ CD44+ TCF-1+ T-BET+	8
PD-1+ LAG-3+ CD38+	7
TIM3+ EOMES+ CD38+	7
PD-1+ TOX1+ CD38+ CD44+ T-BET+	7
PD-1+ TOX1+ TIM3+ CD38+ T-BET+	7
PD-1+ TOX1+ TIM3+ LAG-3+ EOMES+ CD38+ CD44+	7
PD-1+ TOX1+ TIM3+ LAG-3+ EOMES+ CD38+ TCF-1+	7
PD-1+ TOX1+ LAG-3+ CD44+ T-BET+	7
PD-1+ TOX1+ TIM3+ CD39+ EOMES+ CD44+ TCF-1+ T-BET+	7
PD-1+ TOX1+ LAG-3+ CD39+ CD38+	7
PD-1+ TIM3+ LAG-3+ CD39+ EOMES+ CD38+ CD44+ TCF-1+	7
LAG-3+ CD39+ CD38+ TCF-1+	7
PD-1+ TOX1+ LAG-3+ CD39+ EOMES+ CD44+	7
PD-1+ TOX1+ LAG-3+ CD39+ EOMES+ CD44+ TCF-1+ T-BET+	7
PD-1+ TOX1+ LAG-3+ CD39+ EOMES+ CD38+ CD44+ T-BET+	7
PD-1+ TOX1+ EOMES+ CD38+ T-BET+	7
TOX1+ TIM3+ LAG-3+ CD39+ CD38+ T-BET+	7
TOX1+ LAG-3+ CD39+ CD38+ T-BET+	7
TIM3+ LAG-3+ CD38+ CD44+ TCF-1+	7
TOX1+ LAG-3+ EOMES+ CD38+ CD44+ TCF-1+ T-BET+	7
TOX1+ LAG-3+ CD39+ CD44+	7
TIM3+ LAG-3+ CD39+ CD38+ CD44+ TCF-1+ T-BET+	7
TOX1+ TIM3+ LAG-3+ CD38+ TCF-1+ T-BET+	7
TIM3+ LAG-3+ CD39+ EOMES+ TCF-1+	7
TOX1+ CD39+ EOMES+ CD38+ TCF-1+ T-BET+	7
PD-1+ CD38+ CD44+ TCF-1+	7
PD-1+ CD38+ CD44+	7
TOX1+ LAG-3+ CD39+ CD38+ CD44+ TCF-1+	7
TOX1+ TIM3+ LAG-3+ EOMES+ TCF-1+ T-BET+	7
TOX1+ LAG-3+ CD39+ CD38+ CD44+ TCF-1+ T-BET+	7
TIM3+ LAG-3+ CD39+ EOMES+ CD44+ TCF-1+ T-BET+	7
TIM3+ LAG-3+ TCF-1+ T-BET+	7
TOX1+ TIM3+ CD39+ TCF-1+ T-BET+	7
TOX1+ TIM3+ CD39+ T-BET+	7
TIM3+ LAG-3+ EOMES+ CD38+ TCF-1+	7
LAG-3+ EOMES+ CD38+ CD44+ T-BET+	6
TOX1+ TIM3+ LAG-3+ CD39+ CD38+ CD44+ T-BET+	6
PD-1+ TOX1+ LAG-3+ CD39+ EOMES+ CD38+ T-BET+	6
PD-1+ TOX1+ TIM3+ LAG-3+ CD39+ EOMES+ CD38+ T-BET+	6
LAG-3+ CD39+ EOMES+ T-BET+	6
PD-1+ TOX1+ TIM3+ CD39+ EOMES+ TCF-1+ T-BET+	6
TIM3+ LAG-3+ CD39+ EOMES+	6

bioRxiv preprint doi: https://doi.org/10.1101/2023.10.20.563335; this version posted October 23, 2023. The copyright holder for this preprint (which was not certified by peer review) is the author/funder, who has granted bioRxiv a license to display the preprint in perpetuity. It is made available under aCC-BY 4.0 International license.

PD-1+ TOX1+ CD39+ EOMES+ CD38+ TCF-1+ T-BET+	6
PD-1+ LAG-3+ EOMES+ CD38+ TCF-1+ T-BET+	6
PD-1+ TIM3+ LAG-3+ CD39+ EOMES+ TCF-1+ T-BET+	6
TOX1+ TIM3+ LAG-3+ CD44+ TCF-1+ T-BET+	6
TOX1+ TIM3+ LAG-3+ CD39+ EOMES+	6
TOX1+ LAG-3+ CD44+ TCF-1+ T-BET+	6
LAG-3+ CD39+ EOMES+ CD38+ T-BET+	6
PD-1+ TOX1+ TIM3+ LAG-3+ TCF-1+ T-BET+	6
TIM3+ LAG-3+ CD39+ CD38+	6
TOX1+ TIM3+ CD39+ CD38+ CD44+	6
PD-1+ TIM3+ CD44+ TCF-1+	6
TOX1+ LAG-3+ CD39+ CD44+ T-BET+	6
PD-1+ CD39+ CD38+ T-BET+	6
TOX1+ CD39+ CD38+ CD44+ TCF-1+	6
TIM3+ CD39+ CD44+ TCF-1+ T-BET+	6
TIM3+ EOMES+ CD38+ TCF-1+ T-BET+	6
PD-1+ TOX1+ TIM3+ CD39+ T-BET+	6
TOX1+ LAG-3+ EOMES+ CD38+ T-BET+	6
PD-1+ TOX1+ TIM3+ CD39+ TCF-1+	6
PD-1+ LAG-3+ CD44+	6
PD-1+ LAG-3+ CD44+ T-BET+	6
LAG-3+ CD39+ CD44+ TCF-1+ T-BET+	6
PD-1+ TIM3+ EOMES+ CD44+ TCF-1+	6
TOX1+ TIM3+ CD39+ CD44+	6
TIM3+ CD39+ CD38+ T-BET+	6
TOX1+ CD39+ CD38+ CD44+ TCF-1+ T-BET+	6
PD-1+ TOX1+ LAG-3+ CD38+ TCF-1+ T-BET+	6
PD-1+ TOX1+ TIM3+ LAG-3+ CD39+ TCF-1+ T-BET+	6
TIM3+ CD39+ CD38+ TCF-1+ T-BET+	6
PD-1+ CD38+ TCF-1+ T-BET+	6
TOX1+ CD39+ EOMES+ CD38+ T-BET+	6
PD-1+ LAG-3+ CD39+ EOMES+ T-BET+	6
TIM3+ LAG-3+ CD39+ CD38+ T-BET+	6
PD-1+ TOX1+ LAG-3+ EOMES+ CD38+ T-BET+	5
PD-1+ TIM3+ LAG-3+ EOMES+ CD38+ TCF-1+	5
TOX1+ TIM3+ CD39+ CD38+ CD44+ T-BET+	5
TOX1+ TIM3+ LAG-3+ EOMES+ CD38+ T-BET+	5
PD-1+ TOX1+ LAG-3+ EOMES+ CD38+ TCF-1+ T-BET+	5
TOX1+ TIM3+ CD39+ CD38+ T-BET+	5
TOX1+ TIM3+ CD39+ CD38+	5
TOX1+ TIM3+ LAG-3+ CD39+ CD38+	5
PD-1+ TIM3+ EOMES+ TCF-1+ T-BET+	5
PD-1+ TIM3+ EOMES+ CD44+ TCF-1+ T-BET+	5
PD-1+ TOX1+ TIM3+ CD38+ CD44+ TCF-1+	5

bioRxiv preprint doi: https://doi.org/10.1101/2023.10.20.563335; this version posted October 23, 2023. The copyright holder for this preprint (which was not certified by peer review) is the author/funder, who has granted bioRxiv a license to display the preprint in perpetuity. It is made available under aCC-BY 4.0 International license.

TOX1+ LAG-3+ CD39+ CD38+ TCF-1+	5
TOX1+ LAG-3+ CD39+ EOMES+ TCF-1+	5
PD-1+ TOX1+ LAG-3+ CD39+ CD44+	5
PD-1+ TOX1+ LAG-3+ CD39+ CD38+ T-BET+	5
PD-1+ TOX1+ LAG-3+ CD39+ CD38+ TCF-1+ T-BET+	5
TOX1+ TIM3+ CD39+ CD44+ TCF-1+	5
TOX1+ LAG-3+ CD38+ CD44+	5
TOX1+ TIM3+ LAG-3+ CD38+ TCF-1+	5
PD-1+ LAG-3+ CD39+ TCF-1+ T-BET+	5
TOX1+ CD39+ EOMES+ CD38+ CD44+ TCF-1+ T-BET+	5
PD-1+ LAG-3+ CD39+ EOMES+ CD44+ TCF-1+	5
PD-1+ LAG-3+ EOMES+ CD44+	5
PD-1+ EOMES+ CD38+ TCF-1+	5
TIM3+ LAG-3+ CD38+ TCF-1+	5
PD-1+ TOX1+ TIM3+ LAG-3+ CD39+ CD44+	5
TOX1+ TIM3+ LAG-3+ CD38+ CD44+ TCF-1+	5
TOX1+ TIM3+ LAG-3+ EOMES+	5
TIM3+ CD39+ EOMES+ TCF-1+ T-BET+	5
TOX1+ TIM3+ LAG-3+ EOMES+ CD44+ TCF-1+ T-BET+	5
TIM3+ EOMES+ CD38+ CD44+ T-BET+	5
PD-1+ TOX1+ TIM3+ LAG-3+ CD39+ EOMES+	5
PD-1+ TOX1+ TIM3+ LAG-3+ CD39+ EOMES+ TCF-1+ T-BET+	5
PD-1+ CD39+ CD38+ TCF-1+	5
TIM3+ LAG-3+ CD39+ EOMES+ CD38+ CD44+ TCF-1+	5
PD-1+ LAG-3+ CD39+ TCF-1+	5
PD-1+ TOX1+ LAG-3+ CD39+ CD38+ CD44+ T-BET+	5
PD-1+ LAG-3+ CD39+ EOMES+	5
PD-1+ TOX1+ LAG-3+ CD38+ CD44+	5
TOX1+ TIM3+ CD39+ EOMES+ CD44+ TCF-1+	5
TOX1+ TIM3+ LAG-3+ TCF-1+ T-BET+	5
PD-1+ TOX1+ LAG-3+ CD38+ CD44+ TCF-1+ T-BET+	5
PD-1+ TIM3+ LAG-3+ CD39+ CD38+ T-BET+	5
PD-1+ TOX1+ LAG-3+ CD38+ T-BET+	5
TIM3+ LAG-3+ CD38+	5
PD-1+ LAG-3+ CD39+ CD44+ TCF-1+	5
TOX1+ LAG-3+ CD38+ TCF-1+	5
TIM3+ LAG-3+ CD39+ CD38+ TCF-1+ T-BET+	5
PD-1+ TIM3+ LAG-3+ EOMES+ CD38+ CD44+ TCF-1+	4
PD-1+ TIM3+ LAG-3+ T-BET+	4
TOX1+ TIM3+ LAG-3+ CD39+ CD38+ CD44+	4
TIM3+ EOMES+ CD38+ TCF-1+	4
TOX1+ TIM3+ CD39+ EOMES+ TCF-1+ T-BET+	4
TIM3+ EOMES+ CD38+ T-BET+	4
TOX1+ TIM3+ CD39+ CD38+ CD44+ TCF-1+	4

bioRxiv preprint doi: https://doi.org/10.1101/2023.10.20.563335; this version posted October 23, 2023. The copyright holder for this preprint (which was not certified by peer review) is the author/funder, who has granted bioRxiv a license to display the preprint in perpetuity. It is made available under aCC-BY 4.0 International license.

PD-1+ TIM3+ LAG-3+ CD38+ CD44+ TCF-1+ T-BET+	4
TOX1+ TIM3+ CD39+ EOMES+ CD44+ TCF-1+ T-BET+	4
PD-1+ TOX1+ CD39+ CD38+ T-BET+	4
TOX1+ TIM3+ LAG-3+ CD39+ EOMES+ CD38+ CD44+	4
TOX1+ TIM3+ LAG-3+ CD39+ EOMES+ CD38+ CD44+ T-BET+	4
PD-1+ TOX1+ LAG-3+ CD39+ CD38+ CD44+ TCF-1+	4
PD-1+ TIM3+ LAG-3+ CD39+ EOMES+ CD38+ CD44+ T-BET+	4
PD-1+ TIM3+ LAG-3+ EOMES+ CD38+ TCF-1+ T-BET+	4
PD-1+ TIM3+ EOMES+ TCF-1+	4
TIM3+ LAG-3+ EOMES+ CD44+ T-BET+	4
PD-1+ EOMES+ CD38+ CD44+ TCF-1+ T-BET+	4
PD-1+ TOX1+ TIM3+ LAG-3+ CD39+ CD38+ TCF-1+ T-BET+	4
PD-1+ TOX1+ TIM3+ CD39+ CD38+ CD44+	4
TOX1+ LAG-3+ CD38+ CD44+ TCF-1+	4
TOX1+ LAG-3+ CD38+ CD44+ T-BET+	4
PD-1+ CD39+ CD44+	4
PD-1+ LAG-3+ CD39+ CD44+ TCF-1+ T-BET+	4
TIM3+ CD39+ EOMES+ CD44+ TCF-1+	4
PD-1+ LAG-3+ CD44+ TCF-1+ T-BET+	4
PD-1+ TIM3+ TCF-1+ T-BET+	4
PD-1+ EOMES+ CD38+ CD44+ TCF-1+	4
TIM3+ LAG-3+ CD39+ CD38+ CD44+ T-BET+	4
PD-1+ LAG-3+ CD38+ T-BET+	4
PD-1+ TOX1+ TIM3+ LAG-3+ CD39+ CD44+ T-BET+	4
PD-1+ TOX1+ TIM3+ LAG-3+ CD38+ CD44+ TCF-1+	4
PD-1+ TOX1+ TIM3+ LAG-3+ CD39+ TCF-1+	4
PD-1+ TOX1+ TIM3+ LAG-3+ EOMES+ TCF-1+ T-BET+	4
PD-1+ LAG-3+ CD39+ EOMES+ CD38+ CD44+ TCF-1+	4
PD-1+ LAG-3+ CD39+ EOMES+ CD44+ TCF-1+ T-BET+	4
PD-1+ TOX1+ TIM3+ EOMES+ T-BET+	4
TOX1+ LAG-3+ CD39+ CD38+ CD44+	4
PD-1+ TIM3+ CD44+ T-BET+	4
PD-1+ TOX1+ TIM3+ EOMES+ CD44+ TCF-1+ T-BET+	4
TOX1+ TIM3+ CD38+ CD44+ T-BET+	4
TOX1+ LAG-3+ EOMES+ CD38+ CD44+ T-BET+	4
PD-1+ TOX1+ TIM3+ LAG-3+ CD39+ EOMES+ CD44+ T-BET+	4
PD-1+ TOX1+ TIM3+ EOMES+ CD44+ T-BET+	4
PD-1+ TIM3+ CD39+ TCF-1+	4
PD-1+ TOX1+ TIM3+ EOMES+ CD38+ CD44+ TCF-1+	4
PD-1+ TIM3+ EOMES+ CD44+ T-BET+	4
TIM3+ LAG-3+ CD39+ EOMES+ CD38+ CD44+	3
TOX1+ CD39+ CD38+ TCF-1+ T-BET+	3
TIM3+ LAG-3+ CD39+ EOMES+ CD38+ TCF-1+	3
PD-1+ TOX1+ TIM3+ LAG-3+ CD39+ CD38+ CD44+ TCF-1+	3

bioRxiv preprint doi: https://doi.org/10.1101/2023.10.20.563335; this version posted October 23, 2023. The copyright holder for this preprint (which was not certified by peer review) is the author/funder, who has granted bioRxiv a license to display the preprint in perpetuity. It is made available under aCC-BY 4.0 International license.

TOX1+ TIM3+ LAG-3+ CD38+ CD44+ T-BET+	3
TOX1+ TIM3+ LAG-3+ CD39+ TCF-1+ T-BET+	3
PD-1+ TOX1+ EOMES+ CD38+ CD44+ T-BET+	3
TOX1+ TIM3+ LAG-3+ EOMES+ CD44+	3
TIM3+ CD38+ TCF-1+	3
TIM3+ LAG-3+ CD39+ EOMES+ CD38+	3
TOX1+ TIM3+ LAG-3+ EOMES+ CD44+ TCF-1+	3
TIM3+ LAG-3+ CD39+ EOMES+ CD44+ T-BET+	3
PD-1+ TOX1+ TIM3+ LAG-3+ CD39+ CD38+ T-BET+	3
TOX1+ TIM3+ LAG-3+ CD38+ CD44+	3
PD-1+ TOX1+ TIM3+ LAG-3+ CD39+	3
TOX1+ TIM3+ LAG-3+ T-BET+	3
PD-1+ TOX1+ TIM3+ LAG-3+ EOMES+ CD38+ CD44+ T-BET+	3
TIM3+ LAG-3+ CD39+ EOMES+ TCF-1+ T-BET+	3
TIM3+ LAG-3+ EOMES+ CD38+ T-BET+	3
TIM3+ LAG-3+ CD39+ T-BET+	3
TIM3+ LAG-3+ EOMES+ CD44+	3
TIM3+ LAG-3+ CD39+ TCF-1+	3
TIM3+ LAG-3+ CD39+ TCF-1+ T-BET+	3
TOX1+ TIM3+ CD39+ CD44+ T-BET+	3
TIM3+ LAG-3+ CD39+ CD44+ TCF-1+ T-BET+	3
TOX1+ LAG-3+ EOMES+ CD44+ TCF-1+ T-BET+	3
PD-1+ TOX1+ TIM3+ CD39+ CD44+ TCF-1+	3
TIM3+ LAG-3+ CD39+ CD38+ CD44+	3
TOX1+ LAG-3+ CD38+ CD44+ TCF-1+ T-BET+	3
PD-1+ TOX1+ LAG-3+ CD38+ CD44+ TCF-1+	3
PD-1+ TOX1+ TIM3+ CD39+ EOMES+ CD38+ TCF-1+ T-BET+	3
PD-1+ TOX1+ TIM3+ CD39+ EOMES+ CD38+ CD44+	3
LAG-3+ CD38+ CD44+ T-BET+	3
TOX1+ TIM3+ CD39+ EOMES+ CD38+ TCF-1+ T-BET+	3
TOX1+ TIM3+ CD39+ EOMES+ CD38+ CD44+ T-BET+	3
TIM3+ LAG-3+ EOMES+ CD44+ TCF-1+	3
PD-1+ TOX1+ TIM3+ LAG-3+ T-BET+	3
PD-1+ TOX1+ TIM3+ LAG-3+ CD44+ TCF-1+	3
LAG-3+ CD39+ EOMES+ CD38+ CD44+ T-BET+	3
TIM3+ LAG-3+ CD39+ CD38+ CD44+ TCF-1+	3
PD-1+ TOX1+ TIM3+ LAG-3+ EOMES+ T-BET+	3
TOX1+ TIM3+ LAG-3+ CD39+ CD44+ T-BET+	3
PD-1+ LAG-3+ CD39+ EOMES+ CD38+ T-BET+	3
TIM3+ LAG-3+ CD44+ TCF-1+	3
PD-1+ TIM3+ EOMES+ CD38+ TCF-1+	3
TIM3+ CD39+ CD44+ T-BET+	3
PD-1+ LAG-3+ CD39+ EOMES+ CD44+	3
TIM3+ LAG-3+ CD44+ TCF-1+ T-BET+	3

bioRxiv preprint doi: https://doi.org/10.1101/2023.10.20.563335; this version posted October 23, 2023. The copyright holder for this preprint (which was not certified by peer review) is the author/funder, who has granted bioRxiv a license to display the preprint in perpetuity. It is made available under aCC-BY 4.0 International license.

PD-1+ EOMES+ CD38+	3
PD-1+ TIM3+ EOMES+ CD44+	3
TIM3+ CD39+ EOMES+ CD44+ TCF-1+ T-BET+	3
PD-1+ LAG-3+ EOMES+ CD38+	3
PD-1+ LAG-3+ EOMES+ CD38+ TCF-1+	3
PD-1+ TIM3+ EOMES+ T-BET+	3
PD-1+ TIM3+ LAG-3+ CD39+ EOMES+ CD44+ TCF-1+	3
PD-1+ LAG-3+ EOMES+ CD38+ CD44+ TCF-1+	3
PD-1+ TIM3+ LAG-3+ CD39+ EOMES+ TCF-1+	3
PD-1+ LAG-3+ EOMES+ CD38+ CD44+ TCF-1+ T-BET+	3
PD-1+ CD38+ T-BET+	3
TIM3+ CD39+ CD38+ CD44+ TCF-1+ T-BET+	3
PD-1+ TIM3+ LAG-3+ EOMES+ TCF-1+ T-BET+	3
PD-1+ LAG-3+ CD39+ EOMES+ CD38+ TCF-1+	3
PD-1+ TIM3+ LAG-3+ CD39+ CD44+ TCF-1+ T-BET+	3
TIM3+ CD39+ EOMES+ CD38+ CD44+ TCF-1+	3
PD-1+ TIM3+ LAG-3+ EOMES+ CD44+ TCF-1+ T-BET+	3
TIM3+ CD39+ EOMES+ CD38+ CD44+ TCF-1+ T-BET+	3
PD-1+ TIM3+ LAG-3+ CD38+ TCF-1+	3
TOX1+ TIM3+ LAG-3+ CD39+ EOMES+ CD38+ TCF-1+	3
PD-1+ CD38+ CD44+ TCF-1+ T-BET+	3
PD-1+ TIM3+ CD39+ EOMES+ CD44+ TCF-1+	3
PD-1+ CD39+ CD38+	3
PD-1+ CD39+ EOMES+ T-BET+	3
PD-1+ CD39+ EOMES+ CD38+ TCF-1+	3
PD-1+ CD39+ CD38+ TCF-1+ T-BET+	3
TOX1+ TIM3+ LAG-3+ CD39+ EOMES+ TCF-1+ T-BET+	3
PD-1+ CD39+ EOMES+ CD44+ TCF-1+ T-BET+	3
PD-1+ CD39+ EOMES+ CD38+ CD44+ T-BET+	3
PD-1+ TIM3+ CD39+ EOMES+ CD38+ CD44+ TCF-1+ T-BET+	3
PD-1+ CD39+ EOMES+ CD38+ CD44+ TCF-1+ T-BET+	3
PD-1+ TIM3+ CD39+	3
PD-1+ TIM3+ CD39+ CD38+ CD44+ TCF-1+ T-BET+	3
PD-1+ TIM3+ EOMES+ CD38+ CD44+ TCF-1+	3
PD-1+ CD39+ CD44+ T-BET+	3
PD-1+ LAG-3+ CD39+ EOMES+ CD38+	2
PD-1+ TOX1+ TIM3+ CD39+ CD38+ CD44+ TCF-1+	2
TOX1+ LAG-3+ EOMES+ CD44+ T-BET+	2
PD-1+ LAG-3+ CD39+ EOMES+ CD38+ CD44+ T-BET+	2
PD-1+ TIM3+ EOMES+ CD38+ CD44+	2
PD-1+ TIM3+ CD39+ CD38+ T-BET+	2
TIM3+ LAG-3+ EOMES+ CD38+ CD44+ T-BET+	2
PD-1+ TOX1+ TIM3+ EOMES+ CD38+ CD44+	2
PD-1+ TIM3+ EOMES+ CD38+ CD44+ T-BET+	2

bioRxiv preprint doi: https://doi.org/10.1101/2023.10.20.563335; this version posted October 23, 2023. The copyright holder for this preprint (which was not certified by peer review) is the author/funder, who has granted bioRxiv a license to display the preprint in perpetuity. It is made available under aCC-BY 4.0 International license.

PD-1+ TIM3+ CD39+ CD44+ T-BET+	2
TOX1+ LAG-3+ CD39+ EOMES+ CD38+ T-BET+	2
TIM3+ LAG-3+ CD44+ T-BET+	2
TIM3+ LAG-3+ CD39+ CD44+ TCF-1+	2
TOX1+ TIM3+ LAG-3+ CD39+ CD44+ TCF-1+	2
TOX1+ LAG-3+ CD39+ CD38+ CD44+ T-BET+	2
PD-1+ TIM3+ CD38+ CD44+ TCF-1+ T-BET+	2
PD-1+ TOX1+ TIM3+ LAG-3+ CD44+	2
PD-1+ LAG-3+ CD39+ CD38+ CD44+ T-BET+	2
PD-1+ TOX1+ TIM3+ CD39+ EOMES+ CD38+ TCF-1+	2
PD-1+ CD39+ CD38+ CD44+ TCF-1+ T-BET+	2
TIM3+ LAG-3+ CD39+ EOMES+ CD38+ TCF-1+ T-BET+	2
PD-1+ TOX1+ TIM3+ LAG-3+ CD39+ EOMES+ T-BET+	2
PD-1+ CD39+ EOMES+ CD38+ T-BET+	2
TIM3+ LAG-3+ CD39+ EOMES+ CD38+ T-BET+	2
PD-1+ TOX1+ TIM3+ LAG-3+ CD39+ CD38+ CD44+	2
PD-1+ CD39+ EOMES+ CD38+ CD44+ TCF-1+	2
PD-1+ TOX1+ TIM3+ LAG-3+ CD39+ CD38+	2
TIM3+ CD39+ EOMES+ CD44+	2
TIM3+ CD39+ EOMES+ TCF-1+	2
PD-1+ LAG-3+ CD38+ CD44+ TCF-1+	2
TIM3+ LAG-3+ CD39+ EOMES+ CD44+	2
TIM3+ CD39+ EOMES+ T-BET+	2
PD-1+ EOMES+ CD38+ TCF-1+ T-BET+	2
TOX1+ CD39+ EOMES+ CD38+ CD44+ T-BET+	2
PD-1+ TOX1+ TIM3+ LAG-3+ EOMES+ CD44+ TCF-1+	2
PD-1+ TOX1+ TIM3+ LAG-3+ EOMES+ CD44+	2
TIM3+ LAG-3+ CD39+ EOMES+ T-BET+	2
TIM3+ CD39+ EOMES+ CD38+ T-BET+	2
PD-1+ LAG-3+ EOMES+ CD38+ CD44+ T-BET+	2
PD-1+ TOX1+ TIM3+ LAG-3+ CD38+ T-BET+	2
PD-1+ TOX1+ TIM3+ LAG-3+ CD38+	2
PD-1+ TOX1+ TIM3+ LAG-3+ CD44+ T-BET+	2
PD-1+ TIM3+ CD39+ EOMES+ TCF-1+ T-BET+	2
TIM3+ CD39+ EOMES+ CD38+ CD44+	2
PD-1+ TOX1+ TIM3+ LAG-3+ TCF-1+	2
PD-1+ LAG-3+ CD39+ CD38+	2
PD-1+ LAG-3+ CD39+ CD38+ T-BET+	2
PD-1+ TOX1+ TIM3+ CD39+ EOMES+ CD38+ CD44+ T-BET+	2
PD-1+ TOX1+ LAG-3+ CD39+ EOMES+ CD44+ T-BET+	2
PD-1+ CD39+ CD38+ CD44+	2
TIM3+ LAG-3+ EOMES+ CD38+	2
TOX1+ TIM3+ LAG-3+ CD39+ EOMES+ CD44+ TCF-1+	2
PD-1+ CD39+ CD38+ CD44+ T-BET+	2

bioRxiv preprint doi: https://doi.org/10.1101/2023.10.20.563335; this version posted October 23, 2023. The copyright holder for this preprint (which was not certified by peer review) is the author/funder, who has granted bioRxiv a license to display the preprint in perpetuity. It is made available under aCC-BY 4.0 International license.

TOX1+ TIM3+ LAG-3+ EOMES+ CD38+	2
TOX1+ TIM3+ LAG-3+ CD39+ EOMES+ TCF-1+	2
TOX1+ TIM3+ LAG-3+ CD39+ EOMES+ CD44+	2
PD-1+ TOX1+ CD39+ CD38+ TCF-1+	2
PD-1+ TOX1+ CD39+ CD38+ CD44+ T-BET+	2
PD-1+ TIM3+ LAG-3+ EOMES+ CD44+ T-BET+	2
PD-1+ TIM3+ LAG-3+ EOMES+ CD44+ TCF-1+	2
TOX1+ TIM3+ CD39+ EOMES+ T-BET+	2
PD-1+ TOX1+ CD39+ CD38+ CD44+ TCF-1+ T-BET+	2
PD-1+ TIM3+ LAG-3+ CD39+ T-BET+	2
TOX1+ TIM3+ LAG-3+ EOMES+ CD38+ CD44+	2
TOX1+ TIM3+ LAG-3+ EOMES+ T-BET+	2
TOX1+ TIM3+ LAG-3+ CD39+ EOMES+ CD38+ T-BET+	2
TIM3+ CD39+ CD38+ CD44+ T-BET+	2
PD-1+ TIM3+ LAG-3+ CD39+ CD44+ T-BET+	2
PD-1+ TOX1+ LAG-3+ CD38+ CD44+ T-BET+	2
PD-1+ TOX1+ LAG-3+ CD38+ TCF-1+	2
PD-1+ TIM3+ LAG-3+ CD39+ EOMES+ CD38+ T-BET+	2
PD-1+ TIM3+ LAG-3+ CD39+ CD38+ CD44+ TCF-1+	2
TOX1+ TIM3+ LAG-3+ CD44+ TCF-1+	2
PD-1+ TIM3+ LAG-3+ CD39+ EOMES+ T-BET+	2
PD-1+ TIM3+ LAG-3+ EOMES+	2
PD-1+ TIM3+ LAG-3+ EOMES+ T-BET+	2
LAG-3+ CD39+ CD38+ CD44+ T-BET+	2
TOX1+ TIM3+ EOMES+ CD38+ CD44+ T-BET+	2
PD-1+ TIM3+ CD39+ EOMES+ CD44+	2
TOX1+ TIM3+ EOMES+ CD38+	2
TOX1+ TIM3+ EOMES+ CD38+ T-BET+	2
TOX1+ TIM3+ LAG-3+ CD39+ CD38+ TCF-1+	2
PD-1+ TIM3+ CD39+ EOMES+ CD38+ TCF-1+ T-BET+	2
TIM3+ LAG-3+ CD38+ CD44+	2
PD-1+ TIM3+ CD39+ EOMES+ CD38+ CD44+ T-BET+	2
PD-1+ TOX1+ LAG-3+ CD39+ CD38+ CD44+	2
TOX1+ TIM3+ LAG-3+ CD44+	2
PD-1+ TIM3+ LAG-3+ TCF-1+ T-BET+	2
PD-1+ TOX1+ LAG-3+ CD39+ CD44+ T-BET+	2
PD-1+ TIM3+ LAG-3+ CD38+ T-BET+	2
TOX1+ TIM3+ LAG-3+ EOMES+ CD38+ CD44+ T-BET+	2
TOX1+ TIM3+ LAG-3+ CD39+ CD44+	1
TOX1+ TIM3+ LAG-3+ CD39+ CD38+ CD44+ TCF-1+	1
PD-1+ TIM3+ LAG-3+ CD39+ EOMES+ CD38+ CD44+	1
PD-1+ CD39+ EOMES+ CD38+ CD44+	1
PD-1+ TIM3+ LAG-3+ CD39+ EOMES+ CD38+ TCF-1+	1
PD-1+ CD39+ EOMES+ CD38+	1

bioRxiv preprint doi: https://doi.org/10.1101/2023.10.20.563335; this version posted October 23, 2023. The copyright holder for this preprint (which was not certified by peer review) is the author/funder, who has granted bioRxiv a license to display the preprint in perpetuity. It is made available under aCC-BY 4.0 International license.

TOX1+ TIM3+ LAG-3+ EOMES+ CD44+ T-BET+	1
PD-1+ TOX1+ TIM3+ LAG-3+ EOMES+ CD38+	1
PD-1+ LAG-3+ EOMES+ CD38+ T-BET+	1
TIM3+ LAG-3+ CD39+ EOMES+ CD38+ CD44+ T-BET+	1
TOX1+ TIM3+ LAG-3+ CD38+	1
PD-1+ TIM3+ LAG-3+ CD39+ EOMES+ CD44+ TCF-1+ T-BET+	1
PD-1+ CD39+ CD38+ CD44+ TCF-1+	1
TOX1+ TIM3+ LAG-3+ CD44+ T-BET+	1
TOX1+ TIM3+ LAG-3+ CD38+ T-BET+	1
PD-1+ TOX1+ TIM3+ LAG-3+ EOMES+ CD38+ T-BET+	1
PD-1+ TOX1+ TIM3+ LAG-3+ CD38+ TCF-1+	1
TOX1+ TIM3+ LAG-3+ CD39+ T-BET+	1
PD-1+ TOX1+ CD39+ CD38+ TCF-1+ T-BET+	1
PD-1+ EOMES+ CD38+ CD44+ T-BET+	1
PD-1+ TOX1+ CD39+ CD38+ CD44+ TCF-1+	1
PD-1+ LAG-3+ CD38+ CD44+	1
PD-1+ TOX1+ TIM3+ LAG-3+ CD39+ EOMES+ CD44+	1
PD-1+ LAG-3+ CD38+ CD44+ T-BET+	1
PD-1+ TOX1+ TIM3+ LAG-3+ CD39+ T-BET+	1
PD-1+ CD39+ EOMES+ CD44+ T-BET+	1
PD-1+ LAG-3+ EOMES+ CD44+ T-BET+	1
PD-1+ TOX1+ TIM3+ LAG-3+ CD39+ CD38+ TCF-1+	1
TOX1+ TIM3+ LAG-3+ CD39+ EOMES+ CD44+ T-BET+	1
PD-1+ EOMES+ CD38+ CD44+	1
TOX1+ TIM3+ LAG-3+ CD39+ EOMES+ T-BET+	1
TOX1+ TIM3+ LAG-3+ CD39+ EOMES+ CD38+	1
PD-1+ EOMES+ CD38+ T-BET+	1
TOX1+ CD39+ CD38+ CD44+ T-BET+	1
TOX1+ TIM3+ LAG-3+ CD39+ TCF-1+	1
PD-1+ TIM3+ LAG-3+ CD39+ CD38+ TCF-1+	1
PD-1+ TIM3+ LAG-3+ CD39+ EOMES+	1
TIM3+ CD39+ EOMES+ CD38+ TCF-1+ T-BET+	1
TOX1+ TIM3+ CD39+ CD38+ TCF-1+	1
PD-1+ TOX1+ TIM3+ EOMES+ CD38+ TCF-1+ T-BET+	1
PD-1+ TOX1+ TIM3+ EOMES+ CD38+ TCF-1+	1
PD-1+ TIM3+ CD38+	1
PD-1+ TIM3+ CD38+ T-BET+	1
PD-1+ TIM3+ CD38+ TCF-1+	1
PD-1+ TIM3+ CD38+ TCF-1+ T-BET+	1
PD-1+ TIM3+ CD38+ CD44+	1
PD-1+ TIM3+ LAG-3+ CD38+ CD44+ TCF-1+	1
PD-1+ TIM3+ LAG-3+ CD38+ CD44+ T-BET+	1
PD-1+ TIM3+ LAG-3+ CD44+ T-BET+	1
PD-1+ TIM3+ LAG-3+ CD44+	1

bioRxiv preprint doi: https://doi.org/10.1101/2023.10.20.563335; this version posted October 23, 2023. The copyright holder for this preprint (which was not certified by peer review) is the author/funder, who has granted bioRxiv a license to display the preprint in perpetuity. It is made available under aCC-BY 4.0 International license.

PD-1+ TIM3+ LAG-3+ TCF-1+	1
PD-1+ TIM3+ CD39+ T-BET+	1
TIM3+ LAG-3+ CD39+ CD44+ T-BET+	1
PD-1+ TIM3+ CD39+ TCF-1+ T-BET+	1
TIM3+ LAG-3+ CD39+ CD44+	1
TOX1+ LAG-3+ CD39+ EOMES+ CD38+ CD44+ T-BET+	1
PD-1+ TIM3+ CD39+ CD44+	1
LAG-3+ CD39+ CD44+ T-BET+	1
PD-1+ TIM3+ CD39+ CD38+	1
PD-1+ TIM3+ CD39+ CD38+ CD44+ T-BET+	1
TIM3+ LAG-3+ EOMES+ CD38+ CD44+	1
PD-1+ TOX1+ TIM3+ EOMES+ CD38+ CD44+ T-BET+	1
PD-1+ TIM3+ LAG-3+ EOMES+ TCF-1+	1
PD-1+ CD38+ CD44+ T-BET+	1
TOX1+ TIM3+ CD39+ EOMES+ CD38+	1
TOX1+ TIM3+ CD39+ EOMES+ CD38+ CD44+ TCF-1+	1
PD-1+ TIM3+ LAG-3+ CD39+ CD38+ CD44+ TCF-1+ T-BET+	1
PD-1+ TIM3+ LAG-3+ CD39+ CD38+ TCF-1+ T-BET+	1
PD-1+ LAG-3+ CD39+ CD44+	1
PD-1+ LAG-3+ CD39+ CD44+ T-BET+	1
PD-1+ TIM3+ CD39+ EOMES+ TCF-1+	1
PD-1+ LAG-3+ CD39+ CD38+ TCF-1+	1
PD-1+ TIM3+ LAG-3+ CD39+ TCF-1+ T-BET+	1
PD-1+ TOX1+ TIM3+ CD39+ EOMES+ CD38+	1
PD-1+ LAG-3+ CD39+ CD38+ CD44+	1
PD-1+ LAG-3+ CD39+ CD38+ CD44+ TCF-1+	1
PD-1+ TIM3+ LAG-3+ EOMES+ CD44+	1
LAG-3+ CD39+ EOMES+ CD44+ T-BET+	1
TIM3+ CD39+ CD38+ CD44+ TCF-1+	1
TOX1+ TIM3+ CD39+ EOMES+ CD44+ T-BET+	1
TIM3+ LAG-3+ CD39+ CD38+ TCF-1+	1
PD-1+ LAG-3+ CD39+ EOMES+ CD44+ T-BET+	1
PD-1+ TOX1+ TIM3+ CD39+ CD38+ T-BET+	1
PD-1+ TOX1+ TIM3+ CD39+ CD38+	1
PD-1+ TOX1+ TIM3+ CD39+ CD44+ T-BET+	1
PD-1+ TOX1+ TIM3+ CD39+ CD44+	1
PD-1+ LAG-3+ CD39+ EOMES+ CD38+ TCF-1+ T-BET+	1
TOX1+ TIM3+ LAG-3+ CD39+ CD44+ TCF-1+ T-BET+	1

bioRxiv preprint doi: https://doi.org/10.1101/2023.10.20.563335; this version posted October 23, 2023. The copyright holder for this preprint (which was not certified by peer review) is the author/funder, who has granted bioRxiv a license to display the preprint in perpetuity. It is made available under aCC-BY 4.0 International license.

Supplementary Table S5. Raw counts of cell-cell spatial interactions present in the dataset.

Cell-Cell Spatial Interaction	Count
Ki-67- Neoplastic & Ki-67- Neoplastic	2876770
Myeloid cells & Myeloid cells	1480053
Myeloid cells & Ki-67- Neoplastic	465886
B cells & B cells	450530
Ki-67- Neoplastic & Ki-67+ Neoplastic	437638
CD44- CD4 Th & CD44- CD4 Th	226250
Mesenchymal & Mesenchymal	220227
CD44- CD4 Th & B cells	219910
CD44- CD8 T Other & CD44- CD4 Th	206383
Mesenchymal & Ki-67- Neoplastic	202107
Ki-67+ Neoplastic & Ki-67+ Neoplastic	165702
CD44- CD8 T Other & CD44- CD8 T Other	158210
CD44- CD8 T Other & B cells	111164
CD44- CD4 Th & Myeloid cells	107677
Myeloid cells & Mesenchymal	90234
CD44- CD8 T Other & Myeloid cells	89976
B cells & Myeloid cells	62552
Myeloid cells & Ki-67+ Neoplastic	58793
CD44- CD8 T Other & Ki-67- Neoplastic	49618
B cells & Ki-67- Neoplastic	49336
CD44- CD4 Th & Ki-67- Neoplastic	48406
CD44- CD4 Th1 Other & CD44- CD4 Th	42570
CD44- CD8 T Other & CD44- CD4 Th1 Other	34844
CD44- CD4 Th & mTREG	32692
CD44- CD4 Th & CD44+ CD4 Th	31233
CD44- CD4 Th & Mesenchymal	29024
CD44- CD8 T Other & mTREG	27872
CD44+ CD4 Th & B cells	27606
CD44- CD8 T Other & Mesenchymal	26494
CD44+ CD4 Th & CD44+ CD4 Th	24718
CD44+ CD4 Th & Myeloid cells	24454
CD44- CD4 Th1 Other & B cells	24437
CD44+ CD8 T Other & Myeloid cells	23784
mTREG & Myeloid cells	23570
CD44- CD8 T Other & CD44+ CD8 T Other	23249
Naive TREG & B cells	23177
CD44- CD4 Th & Naive TREG	22405
CD44+ CD8 T Other & CD44+ CD8 T Other	22351
Mesenchymal & Ki-67+ Neoplastic	20910
CD44+ CD8 T Other & CD44+ CD4 Th	20823
CD44+ CD8 T Other & CD44- CD4 Th	20030
CD44+ CD8 T Other & B cells	19305
CD44- CD8 T Other & Naive TREG	16461

bioRxiv preprint doi: https://doi.org/10.1101/2023.10.20.563335; this version posted October 23, 2023. The copyright holder for this preprint (which was not certified by peer review) is the author/funder, who has granted bioRxiv a license to display the preprint in perpetuity. It is made available under aCC-BY 4.0 International license.

B cells & Mesenchymal	15010
mTREG & B cells	14656
mTREG & Ki-67- Neoplastic	14362
CD44- CD8 T Other & CD44+ CD4 Th	12734
CD44- CD4 Th1 Other & CD44- CD4 Th1 Other	11730
mTREG & Mesenchymal	10419
CD44- CD4 Th1 Other & Myeloid cells	9616
mTREG & mTREG	9087
CD8 T NAIVE & CD44- CD8 T Other	8303
Naive TREG & Naive TREG	7839
CD8 T NAIVE & CD44- CD4 Th	7103
B cells & Ki-67+ Neoplastic	6512
CD44- CD4 Th & Ki-67+ Neoplastic	6452
CD44- CD8 T Other & Ki-67+ Neoplastic	6392
CD44- CD4 Th & T-BET+ TREG	6102
Naive TREG & Myeloid cells	6035
CD44+ CD4 Th & mTREG	5918
CD44- CD4 Th1 Other & Mesenchymal	5711
CD44- CD4 Th1 Other & Ki-67- Neoplastic	5640
CD8 TEMRA & CD44+ CD4 Th	5367
CD44- CD8 T Other & T-BET+ TREG	5300
CD44+ CD8 T Other & mTREG	5186
Naive TREG & Ki-67- Neoplastic	5185
CD8 T NAIVE & B cells	5149
T-BET+ TREG & B cells	5086
Naive TREG & mTREG	4921
CD44+ CD8 T Other & Ki-67- Neoplastic	4863
CD8 TEMRA & CD44+ CD8 T Other	4772
CD44+ CD4 Th & Mesenchymal	4593
CD44- CD4 Th1 Other & mTREG	4305
CD44+ CD4 Th & Naive TREG	4166
CD8 TEMRA & B cells	3878
CD44+ CD8 T Other & Mesenchymal	3536
CD44+ CD4 Th & Ki-67- Neoplastic	3476
CD8 TEMRA & CD44- CD8 T Other	3454
CD8 TEMRA & CD44- CD4 Th	3399
CD44- CD4 Th1 Other & T-BET+ TREG	3382
CD8 TEMRA & CD8 TEMRA	3337
CD8 TEMRA & Myeloid cells	3226
CD44- CD4 Th1 Other & CD44+ CD4 Th	3112
CD44- CD4 Th1 Other & Naive TREG	3052
CD44+ CD8 T Other & CD44- CD4 Th1 Other	2766
CD44+ CD8 T Other & Naive TREG	2300
CD4 Th1EMRA & CD44- CD4 Th	1997

bioRxiv preprint doi: https://doi.org/10.1101/2023.10.20.563335; this version posted October 23, 2023. The copyright holder for this preprint (which was not certified by peer review) is the author/funder, who has granted bioRxiv a license to display the preprint in perpetuity. It is made available under aCC-BY 4.0 International license.

CD4 Th1EM & CD44- CD4 Th	1949
mTREG & Ki-67+ Neoplastic	1924
T-BET+ TREG & Myeloid cells	1857
Naive TREG & Mesenchymal	1852
CD4 Th1EMRA & B cells	1839
Naive TREG & T-BET+ TREG	1681
CD4 Th1EM & Myeloid cells	1667
CD4 Th1EMRA & CD44+ CD4 Th	1607
CD4 Th1EM & CD44+ CD4 Th	1542
CD44- CD8 T Other & CD4 Th1EM	1486
mTREG & T-BET+ TREG	1458
CD8 TEM & CD44- CD8 T Other	1322
CD44+ CD8 T Other & CD4 Th1EM	1311
CD8 TEM & CD44+ CD8 T Other	1285
CD8 TEM & CD44+ CD4 Th	1283
CD8 T NAIVE & CD44- CD4 Th1 Other	1238
CD44- CD8 T Other & CD4 Th1EMRA	1162
CD8 TEM & CD44- CD4 Th	1140
T-BET+ TREG & T-BET+ TREG	1119
CD4 Th1EM & CD44- CD4 Th1 Other	1112
CD44+ CD4 Th & T-BET+ TREG	1092
CD8 TEX & CD44+ CD8 T Other	1071
CD44+ CD8 T Other & T-BET+ TREG	1020
T-BET+ TREG & Ki-67- Neoplastic	1013
T-BET+ TREG & Mesenchymal	969
CD44+ CD8 T Other & CD4 Th1EMRA	969
CD4 Th1EMRA & CD44- CD4 Th1 Other	934
CD4 Th1EMRA & Myeloid cells	932
CD8 TEMRA & Ki-67- Neoplastic	878
CD8 TEM & Myeloid cells	847
CD8 T NAIVE & CD44+ CD8 T Other	837
CD8 TEM & B cells	808
CD4 Th1EM & Mesenchymal	790
CD8 TEMRA & Naive TREG	769
CD8 TEX & CD44- CD8 T Other	754
CD8 T NAIVE & Myeloid cells	740
CD4 Th1EMRA & CD4 Th1EMRA	739
CD44- CD4 Th1 Other & Ki-67+ Neoplastic	727
CD44+ CD8 T Other & Ki-67+ Neoplastic	710
CD4 Th1EM & mTREG	685
CD8 T NAIVE & CD8 T NAIVE	678
CD8 TEM & CD8 TEM	628
CD8 T NAIVE & Naive TREG	618
CD4 Th1EM & B cells	609

bioRxiv preprint doi: https://doi.org/10.1101/2023.10.20.563335; this version posted October 23, 2023. The copyright holder for this preprint (which was not certified by peer review) is the author/funder, who has granted bioRxiv a license to display the preprint in perpetuity. It is made available under aCC-BY 4.0 International license.

CD44+ CD4 Th1 Other & Ki-67- Neoplastic	580
CD8 TEMRA & CD4 Th1EMRA	576
CD8 T NAIVE & mTREG	574
CD44+ CD4 Th1 Other & Myeloid cells	550
CD8 TEX & CD44+ CD4 Th	522
CD8 TEX & CD44- CD4 Th	514
CD4 Th1EM & Ki-67- Neoplastic	510
CD4 Th1EMRA & Naive TREG	507
CD44+ CD4 Th & Ki-67+ Neoplastic	495
CD8 TEM & CD8 TEMRA	478
CD44- CD4 Th1 Other & CD44+ CD4 Th1 Other	477
CD4 Th1EMRA & T-BET+ TREG	476
CD8 TEMRA & CD44- CD4 Th1 Other	467
CD8 TEMRA & mTREG	459
CD8 T NAIVE & CD44+ CD4 Th	457
Naive TREG & Ki-67+ Neoplastic	451
CD8 TEX & B cells	442
CD4 Th1EM & CD4 Th1EM	431
CD8 TEMRA & Mesenchymal	383
CD8 TEX & CD8 TEX	368
CD44+ CD4 Th1 Other & B cells	353
CD8 TEMRA & T-BET+ TREG	350
CD8 TEM & CD4 Th1EM	340
CD44+ CD4 Th1 Other & CD44+ CD4 Th1 Other	338
CD8 TEX & mTREG	316
CD8 TEX & Myeloid cells	316
CD8 TEM & mTREG	309
CD44+ CD4 Th1 Other & CD44- CD4 Th	303
CD4 Th1EMRA & CD44+ CD4 Th1 Other	290
CD8 TTEX & CD44+ CD8 T Other	285
CD4 Th1EM & T-BET+ TREG	281
CD44+ CD4 Th1 Other & Mesenchymal	278
CD8 TEMRA & CD8 TEX	250
CD4 Th1EMRA & Ki-67- Neoplastic	247
CD4 Th1EMRA & mTREG	239
CD8 TTEX & Myeloid cells	221
CD44- CD8 T Other & CD44+ CD4 Th1 Other	215
CD8 T NAIVE & T-BET+ TREG	213
CD44+ CD4 Th1 Other & CD44+ CD4 Th	206
CD8 TEM & CD44- CD4 Th1 Other	199
CD8 TEX & Ki-67- Neoplastic	199
CD8 TTEX & CD44- CD8 T Other	194
CD8 T NAIVE & Mesenchymal	193
CD44+ CD8 T Other & CD44+ CD4 Th1 Other	186

bioRxiv preprint doi: https://doi.org/10.1101/2023.10.20.563335; this version posted October 23, 2023. The copyright holder for this preprint (which was not certified by peer review) is the author/funder, who has granted bioRxiv a license to display the preprint in perpetuity. It is made available under aCC-BY 4.0 International license.

CD8 TTEX & CD44+ CD4 Th	176
CD8 TEMRA & CD4 Th1EM	174
CD4 Th1EMRA & Mesenchymal	172
CD8 TTEX & CD44- CD4 Th	168
CD8 TEM & T-BET+ TREG	159
T-BET+ TREG & Ki-67+ Neoplastic	150
CD8 T NAIVE & CD8 TEMRA	150
CD4 Th1EM & CD4 Th1EMRA	146
CD4 Th1EM & Naive TREG	137
CD8 TEM & CD4 Th1EMRA	131
CD8 T NAIVE & Ki-67- Neoplastic	116
CD8 TTEX & B cells	111
CD8 TEFF & CD44+ CD8 T Other	108
CD8 TEX & Naive TREG	101
CD8 TEX & CD44- CD4 Th1 Other	96
CD8 TEM & CD8 TEX	91
CD8 TEFF & B cells	88
CD8 TEM & Mesenchymal	85
CD8 TEX & T-BET+ TREG	83
CD8 TEX & CD8 TTEX	83
CD8 TEM & Naive TREG	82
CD8 TEMRA & Ki-67+ Neoplastic	81
CD8 TTEX & mTREG	80
CD8 TTEX & Ki-67- Neoplastic	77
CD8 TEX & Mesenchymal	75
CD8 TEX & CD4 Th1EM	72
CD8 TEM & Ki-67- Neoplastic	68
CD44+ CD4 Th1 Other & Ki-67+ Neoplastic	65
CD8 TEFF & CD44- CD8 T Other	64
CD44+ CD4 Th1 Other & T-BET+ TREG	61
CD8 TEFF & CD8 TEMRA	56
CD4 Th1EFF & B cells	56
CD44+ CD4 Th1 Other & mTREG	54
CD8 TEFF & Myeloid cells	50
CD8 TEFF & CD44- CD4 Th	49
CD4 Th1EFF & Myeloid cells	47
CD4 Th1EM & Ki-67+ Neoplastic	45
CD8 TEFF & CD8 TEFF	42
CD4 Th1EM & CD44+ CD4 Th1 Other	41
CD8 T NAIVE & CD4 Th1EMRA	35
CD8 TEMRA & CD8 TTEX	34
CD44+ CD4 Th1 Other & Naive TREG	34
CD8 TEMRA & CD44+ CD4 Th1 Other	31
CD8 TTEX & Mesenchymal	30

bioRxiv preprint doi: https://doi.org/10.1101/2023.10.20.563335; this version posted October 23, 2023. The copyright holder for this preprint (which was not certified by peer review) is the author/funder, who has granted bioRxiv a license to display the preprint in perpetuity. It is made available under aCC-BY 4.0 International license.

CD8 TEX & CD4 Th1EMRA	29
CD8 T NAIVE & CD4 Th1EM	28
CD4 Th1EFF & CD44+ CD4 Th	28
CD8 TEFF & CD44- CD4 Th1 Other	26
CD8 TEFF & Mesenchymal	25
CD8 TTEX & Naive TREG	25
CD8 TEFF & Ki-67- Neoplastic	24
CD4 Th1EMRA & Ki-67+ Neoplastic	23
CD8 T NAIVE & CD8 TEM	22
CD8 TEX & Ki-67+ Neoplastic	22
CD4 Th1EFF & CD44+ CD4 Th1 Other	21
CD8 TTEX & CD44- CD4 Th1 Other	21
CD8 T NAIVE & CD8 TEX	20
CD8 T NAIVE & CD44+ CD4 Th1 Other	20
CD4 Th1EFF & Ki-67- Neoplastic	19
CD4 Th1EFF & CD44- CD4 Th1 Other	18
CD8 TTEX & CD4 Th1EM	18
CD8 TTEX & CD8 TTEX	17
CD8 TTEX & Ki-67+ Neoplastic	15
CD4 Th1EFF & CD4 Th1EMRA	15
CD8 TEFF & CD44+ CD4 Th	13
CD8 TEM & CD8 TTEX	11
CD8 TEMRA & CD4 Th1EFF	11
CD8 TEX & CD44+ CD4 Th1 Other	10
CD44- CD8 T Other & CD4 Th1EFF	8
CD8 T NAIVE & Ki-67+ Neoplastic	8
CD4 Th1EFF & Ki-67+ Neoplastic	8
CD44+ CD8 T Other & CD4 Th1EFF	7
CD4 Th1EFF & CD44- CD4 Th	7
CD8 TTEX & CD4 Th1EMRA	7
CD8 TTEX & T-BET+ TREG	7
CD8 TEM & Ki-67+ Neoplastic	5
CD4 Th1EFF & Naive TREG	5
CD8 TEFF & CD8 TTEX	5
CD8 TEFF & T-BET+ TREG	4
CD8 TEFF & CD44+ CD4 Th1 Other	4
CD8 TEM & CD44+ CD4 Th1 Other	4
CD4 Th1EFF & CD4 Th1EM	4
CD8 TEX & CD4 Th1EFF	4
CD4 Th1EFF & Mesenchymal	4
CD8 TEFF & Naive TREG	4
CD8 T NAIVE & CD8 TEFF	3
CD4 Th1EFF & CD4 Th1EFF	3
CD4 Th1EFF & T-BET+ TREG	3

bioRxiv preprint doi: https://doi.org/10.1101/2023.10.20.563335; this version posted October 23, 2023. The copyright holder for this preprint (which was not certified by peer review) is the author/funder, who has granted bioRxiv a license to display the preprint in perpetuity. It is made available under aCC-BY 4.0 International license.

CD4 Th1EFF & mTREG	2
CD8 T NAIVE & CD8 TTEX	2
CD8 TEM & CD4 Th1EFF	2
CD8 TEFF & mTREG	1
CD8 TEFF & CD8 TEM	1
